# Glabridin inhibited the spread of polymyxin-resistant Enterobacterium carrying ICE*Mmo*MP63

**DOI:** 10.3389/fmicb.2023.1188900

**Published:** 2023-05-22

**Authors:** Jiafang Fu, Yayu Liu, Fengtian Wang, Gongli Zong, Zhen Wang, Chuanqing Zhong, Guangxiang Cao

**Affiliations:** ^1^First Affiliated Hospital of Shandong First Medical University, Biomedical Sciences College & Shandong Medicinal Biotechnology Centre, Shandong First Medical University & Shandong Academy of Medical Sciences, Jinan, China; ^2^National Health Commission (NHC) Key Laboratory of Biotechnology Drugs. Shandong Academy of Medical Sciences, Jinan, China; ^3^Jinan Municipal Minzu Hospital, Jinan, China; ^4^School of Municipal and Environmental Engineering, Shandong Jianzhu University, Jinan, China

**Keywords:** *Morganella morganii*, polymyxin resistance, integrative and conjugative element, MFS transporter, glabridin

## Abstract

**Introduction:**

The role of integrative and conjugative elements (ICEs) in antibiotic resistance in *Morganella morganii* is unknown. This study aimed to determine whether an ICE identified in the *M. morganii* genome contributed to the polymyxin resistance.

**Methods:**

Whole-genome sequencing was performed followed by bioinformatics analyses to identify ICEs and antibiotic resistance genes. Conjugation assays were performed to analyze the transferability of a discovered ICE. A drug transporter encoded on the ICE was heterogeneously expressed in *Escherichia coli*, minimum inhibitory concentrations of antibiotics were determined, and a traditional Chinese medicine library was screened for potential efflux pump inhibitors.

**Results:**

An antibiotic resistance-conferring ICE, named ICE*Mmo*MP63, was identified. ICE*Mmo*MP63 was verified to be horizontally transferred among Enterobacteriaceae bacteria. G3577_03020 in ICE*Mmo*MP63 was found to mediate multiple antibiotic resistances, especially polymyxin resistance. However, natural compound glabridin was demonstrated to inhibit polymyxin resistance.

**Discussion:**

Our findings support the need for monitoring dissemination of ICE*Mmo*MP63 in Enterobacteriaceae bacteria. Combined glabridin and polymyxin may have therapeutic potential for treating infections from multi-drug resistant bacteria carrying ICE*Mmo*MP63.

## Introduction

Antibiotic-resistant, especially multiple antibiotic-resistant (MAR), pathogens have become a serious threat to human health (Davies et al., 2013; He et al., 2016). Antibiotic resistance genes (ARGs) from MAR bacteria can be considered a type of genetic pollution in the environment (Pruden et al., 2006), and therefore, horizontal transfer of ARGs among MAR bacteria is an important topic in both the environmental sciences and medical sciences. Transduction (Thierauf et al., 2009), transformation (Kruger and Stingl, 2011), conjugation (Lederberg and Tatum, 1946), and fusion of cells with DNA-containing vesicles or fusion of two cells (Johnson and Grossman, 2015) are four ways for bacteria to horizontally transfer ARGs, although conjugation via integrative and conjugative elements (ICEs) is considered to be the more common mode (Guglielmini et al., 2011). ICEs, which can range from about 20 to 500 kb in size, are mobile genetic elements that are integrated into bacterial chromosomes and that can excise and be transferred to new cells (Carraro and Burrus, 2015; Burrus, 2017; de Assis et al., 2022). Typical ICEs are integrated into specific sites (such as 3′-ends of tRNA/tmRNA genes and 3′-end of the guanosine monophosphate synthetase-encoding gene *guaA*) in a host genome and encode a type IV conjugation system, which enables an ICE to transfer to other bacteria via conjugation (Alvarez-Martinez and Christie, 2009; Del Canto et al., 2011; Song et al., 2012; Christie et al., 2014; Trokter et al., 2014). ICEs encoding ARGs can endow the recipient bacteria with antibiotic resistance, which is of particular concern when the recipient bacteria is a human pathogen as this makes eradication more difficult, potentially leading to disease outbreaks from the pathogen. However, clinical pathogens are not always screened for ICEs encoding ARGs, which is detrimental to the prevention and treatment of MAR bacteria.

*Morganella morganii* is an increasingly important opportunistic pathogen because of its virulence and increased antibiotic resistance and because it can cause a variety of clinical infections, such as urinary tract infections, wound infections, endocarditis, septic shock, osteomyelitis, bacteremia and sepsis, acute postoperative endophthalmitis, pericarditis, intra-abdominal abscess, peritonitis, rhabdomyolysis, orbital abscess, black nail infection, and shoulder septic arthritis (Casanova-Roman et al., 2002; Liu et al., 2016, 2021; Tsiotsias et al., 2019; Minnullina et al., 2022; Agrawal et al., 2023). *M. morganii* is a rod-shaped, Gram-negative bacillus that belongs to the tribe Proteeae of the Enterobacteriaceae family and that is found in intestinal tracts of humans, mammals, and reptiles and in the environment (Liu et al., 2016; Erlanger et al., 2019). In rare cases, especially in hospital and postoperative environments, and in patients and young children with impaired immune systems, it can cause potentially fatal systemic infections (Bandy, 2020). *M. morganii* was reported to be resistant to many antibiotics, including tetracyclines, macrolides, glycopeptides, rifampicin, lincosamides, streptogramins, daptomycin, colistin, fusidic acid, and nitrofurantoin (Leclercq et al., 2013; Seija et al., 2015), indicating this species carries many ARGs in the genome and acts as an ARG reservoir. Plasmid-mediated quinolone-resistance has also been reported in *M. morganii* (Mahrouki et al., 2012). However, no ICEs carrying ARGs have been reported in *M. morganii*.

In this study, the MAR strain *M. morganii* MP63 was isolated from a hospital sewage sample. A typical ICE associated with multiple antibiotic resistances was identified in the MP63 genome and named ICE*Mmo*MP63. We found that ICE*Mmo*MP63 could horizontally transfer to *Escherichia coli* strains, indicating that this ICE could disseminate ARGs among Enterobacteriaceae bacteria, raising the need to monitor and inhibit ICE*Mmo*MP63 in order to prevent further outbreaks of infection.

## Materials and methods

### Isolation and identification of strain MP63

MP63 strain was isolated from a sewage sample from a hospital in Jinan, China. The sewage sample was serially diluted 10-fold with sterilized water, plated onto Luria Bertani (LB) solid medium supplemented with 16 μg/ml polymyxin E, and the plates were incubated overnight at 28°C to obtain single colonies. Then, a selected single colony was streaked three consecutive times on LB solid medium supplemented with polymyxin E to obtain a pure culture. After streaking and purification, a selected pure culture was named strain MP63.

The 16S rDNA gene of MP63 was amplified by PCR using the universal primers 27F and 1492R (Supplementary Table 1), and then PCR products were purified using PCR Clean Up Kit (Beyotime, China) and sequenced at BioSune Co., Ltd (Shanghai, China). The 16S rDNA sequence was analyzed using BLAST^1^ for preliminary identification.

### Antibiotic minimum inhibitory concentration (MIC) testing

The minimum inhibitory concentrations (MICs) of antibiotics for strain MP63 were determined using the broth microdilution method as previously described (CLSI, 2017). The transconjugant 25DN-MP and recombinant strain M3020 were also tested for MICs. All MIC tests in this study were carried out in triplicate.

### Whole-genome sequencing and genomic analysis

The MP63 genome was sequenced using the Nanopore and BGISEQ-500 platform (BGI, Wuhan, China) and assembled using Unicycler software (Wick et al., 2017). Genome annotation was performed using the NCBI website,^2^ the RASTtk server (Overbeek et al., 2014; Brettin et al., 2015), and the Pathosystems Resource Integration Center (PATRIC) server (Wattam et al., 2017). Antibiotic resistance genes were predicted using ARDB (Antibiotic Resistance Genes Database) (Liu and Pop, 2009), ARG-ANNOT (Antibiotic Resistance Gene-ANNOTation database) (Gupta et al., 2014), and CARD (the Comprehensive Antibiotic Research Database) (Alcock et al., 2020). The virulence factors in the MP63 genome were predicted using VFDB (virulence factor database) (Liu B. et al., 2019). Sequence alignment was performed using the BLAST server and UniProt server.^3^

### Phylogenetic analysis of strain MP63

Phylogenetic analysis was performed using the MP63 genome sequence, and a whole-genome phylogenetic tree was constructed using the PATRIC server (Wattam et al., 2017) and 12 genome sequences belonging to Morganellaceae species.

### Identification and annotation of ICE*Mmo*MP63

Genomic islands in MP63 genome were predicted by IslandViewer 4 (Bertelli et al., 2017). An identified ICE was further analyzed using ICEberg 2.0 software (Liu M. et al., 2019) and named ICE*Mmo*MP63. ICE*Mmo*MP63 genes were annotated using NCBI and the RASTtk server (Overbeek et al., 2014; Brettin et al., 2015), and insertion sequences were predicted using IS-Finder (Siguier et al., 2012).

### Phylogenetic relationships of ICE*Mmo*MP63

Alignment of the whole nucleotide sequence of ICE*Mmo*MP63 was performed using the BLAST server, and a phylogenetic tree was constructed using MEGA7 software (Kumar et al., 2016).

### Conjugation assays

Horizontal transferability of ICE*Mmo*MP63 was tested using conjugation assays as previously described with some modifications (Fu et al., 2020). Strain MP63 and *E. coli* 25DN were used as the donor and recipient strains, respectively. MP63 and 25DN were mixed together and cultured on LB solid medium with polymyxin E (32 mg/L), sodium azide (1.7 mol/L), and X-Gluc (5-bromo-4-chloro-3-indolyl-beta-D-glucuronic acid) to screen the transconjugants. The presence of ICE*Mmo*MP63 in the transconjugants was demonstrated by PCR using primer pairs Val-F and Val-R (Supplementary Table 1) and DNA sequencing.

### Construction of recombinant strain M3020 of G3577_03020

Gene G3577_03020, encoding a major facilitator superfamily (MFS) transporter, was amplified using primers M3020-F/R (Supplementary Table 1) and MP63 genomic DNA as template, and the β-lactamase gene promoter was amplified using primers AP-F/R (Supplementary Table 1) and pMD18-T vector as template. These two amplicons were then fused by PCR using primers AP-F and M3020-R (Supplementary Table 1), followed by cloning into the pMD18-T vector to obtain the recombinant pMD18-3020. After verification by DNA sequencing, pMD18-3020 was transformed into *E. coli* DH5α (TSINGKE, China) for heterogeneous expression of G3577_03020, and the recombinant strain was named M3020.

### Determination of MICs of strain M3020 with and without efflux pump inhibitors

Antibiotic MICs for M3020 were determined using the broth microdilution method as described above. The MICs for polymyxin E, tetracycline, and cefixime were also calculated by broth microdilution in the presence of efflux pump inhibitors (EPIs), including carbonyl cyanide m-chlorophenylhydrazone (CCCP), N-methyl-2-pyrrolidone (NMP), reserpine (RES), and verapamil (VER). CCCP, NMP, RES, and VER were used at final concentrations of 0.1, 8, 8, and 8 mg/L, respectively. *E. coli* DH5α strain harboring pMD18-T was used in each test for internal quality control.

The Traditional Chinese Medicine Active Compound Library (MedChemExpress, China) was screened for potential EPIs of the MFS transporter G3577_03020. The MICs for polymyxin E, tetracycline, and cefixime in the presence of traditional Chinese medicine compounds were determined as above. Each traditional Chinese medicine compound was used at final concentrations of 5, 15, and 25 μM. *E. coli* DH5α strain harboring pMD18-T was used in each test for internal quality control.

### Homology modeling and molecular docking

Protein homology modeling and molecular docking were conducted as previously described with some modifications (Zong et al., 2020). The homology model for the MFS transporter was constructed using the Swiss model (Waterhouse et al., 2018). The small molecule ligands of polymyxin E, cefixime, tetracycline, and EPIs were drawn using Discovery Studio. The molecular docking was carried out according to the CDOCKER protocol of Discovery Studio 2.0 (Biovia, 2017) to determine whether the above small molecules could bind G3577_03020.

## Results

### Antibiotic-resistant phenotype and genomic features of strain MP63

**FIGURE 1:**
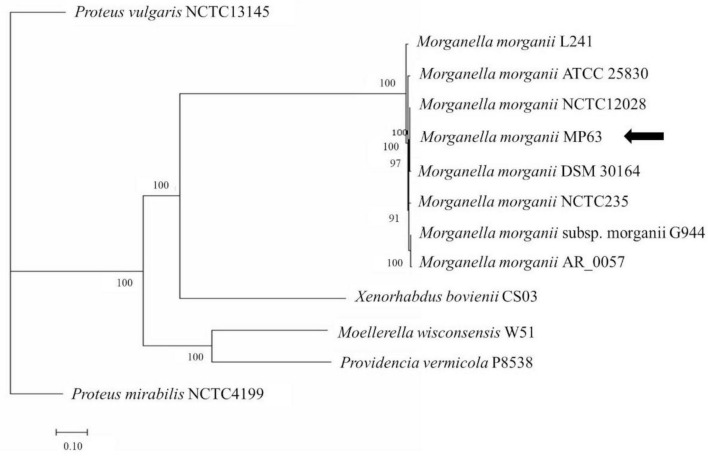
Molecular phylogenetic analysis of MP63 based on genome sequence. Twelve strains from the family Morganellaceae were selected to analyze the phylogenetic relationship of MP63. *Proteus mirabilis* NCTC4199 and *Proteus vulgaris* NCTC13145 belong to the genus *Proteus*, *Providencia vermicola* P8538 belongs to the genus *Providencia*, *Moellerella wisconsensis* W51 belongs to the genus *Moellerella*, *Xenorhabdus bovienii* CS03 belongs to the genus *Xenorhabdus*, and the other seven strains (L241, ATCC 25830, NCTC12028, DSM 30164, NCTC235, G944, AR_0057) belong to the genus *Morganella* in the family Morganellaceae. The whole-genome phylogenetic tree was constructed using the PATRIC server. MP63 is most closely related to *Morganella morganii* NCTC12028. The position of MP63 in the phylogenetic tree is indicated by the black arrow.

Together with the preliminary 16S rDNA gene analysis and alignment of the whole genome sequence, phylogenetic tree analysis revealed that strain MP63 is most closely related to strain *M. morganii* NCTC12028 (Figure 1), and therefore strain MP63 was identified as *M. morganii*. MIC assays revealed that strain MP63 was resistant to five tested antibiotics (Supplementary Table 2), including polymyxin E (MIC > 128 mg/L), cefixime (MIC > 128 mg/L), meropenem (MIC = 32 mg/L), florfenicol (MIC = 16 mg/L), and tetracycline (MIC = 96 mg/L), indicating that *M. morganii* MP63 was a MAR strain.

To understand the basis for its antibiotic resistance profile and its pathogenicity, the whole genome of *M. morganii* MP63 was sequenced and analyzed. MP63 contains one circular chromosome, which is 4,004,672 bp in size, with an average GC content of 51.1%. MP63 also contains the three plasmids pMP63A, pMP63B, and pMP63C. Genome annotation identified 3,807 genes in the MP63 genome, including 3,661 protein-coding genes (Supplementary Figure 1 and Supplementary Table 3). Consistent with its antibiotic resistance profile, 42 antibiotic resistance-associated genes were found in the MP63 genome (Supplementary Table 4). Additionally, based on the VFDB database, the genome contained 321 putative virulence factor-encoding genes (Supplementary Table 5), suggesting that MP63 is potentially pathogenic for humans or other animals.

### Identification of the integrative and conjugative element ICE*Mmo*MP63

**FIGURE 2:**
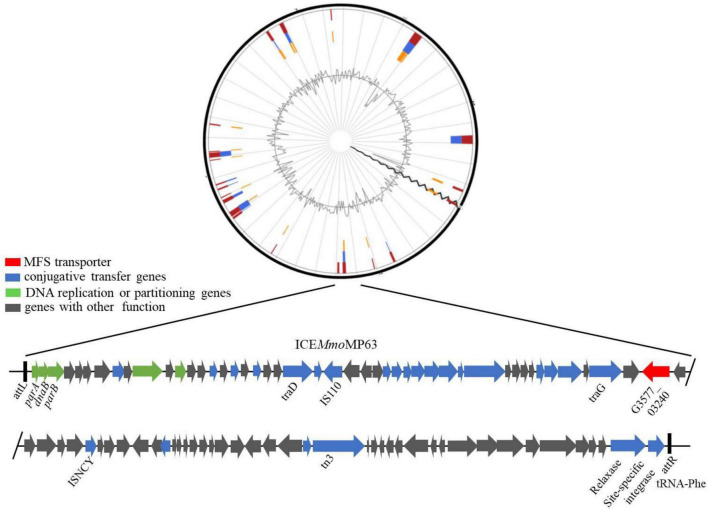
Schematic view of the integrative and conjugative element ICE*Mmo*MP63. **Top image**: predicted position of ICE*Mmo*MP63 in the MP63 genome using IslandViewer 4. **Bottom image**: gene arrangement and characteristics of ICE*Mmo*MP63 identified using ICEberg 2.0. ICE*Mmo*MP63 is bordered by a 21-bp DR (5′-GACACGGGGATTTTCAATCCC-3′), indicated by black bars, in the chromosome of MP63.

IslandViewer 4 analysis revealed several genomic islands in MP63 genome, and further analysis using ICEberg 2.0 identified a novel ICE, which was named ICE*Mmo*MP63 (Figure 2). ICE*Mmo*MP63 extended from position 604,119 to 693,424 in the MP63 genome and contained 89,306 bp. ICE*Mmo*MP63 was bordered by a 21-bp direct repeat (DR) (5′-GACACGGGGATTTTCAATCCC-3′) at both ends and was inserted into the *tRNA-Phe* gene (G3577_03240). Gene annotation revealed that ICE*Mmo*MP63 contained 90 open reading frames (ORFs), including an MFS transporter-encoding gene G3577_03020, the function of which was unclear and was not included in the annotated antibiotic resistance genes in Supplementary Table 4. ICE*Mmo*MP63 also contained multiple genes associated with type IV conjugative transfer systems (Supplementary Table 6), suggesting that ICE*Mmo*MP63 encoded such a system.

### Phylogenetic relationship of ICE*Mmo*MP63

**FIGURE 3:**
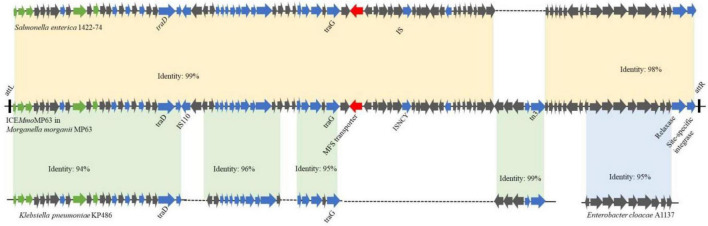
Schematic showing potential sources of genes in ICE*Mmo*MP63. Gene sequences in ICE*Mmo*MP63 were compared with gene sequences from *Salmonella enterica* 1422-74, *Klebsiella pneumoniae* KP486, and *Enterobacter cloacae* A1137.

As ICE*Mmo*MP63 has characteristics typical of ICEs and might have been horizontally transferred from other bacteria, the phylogenetic relationship of ICE*Mmo*MP63 was further analyzed. Eight related mobile element sequences from different strains based on the BLASTn alignment results were chosen, and the phylogenetic tree revealed that ICE*Mmo*MP63 was most closely related to mobile elements in Enterobacterales species (Supplementary Figure 2). Pairwise alignment using BLAST and the whole ICE*Mmo*MP63 nucleotide sequence showed that ICE*Mmo*MP63 has strong homology with the mobile elements *Salmonella enterica* 1422-74 (Identity: 99%), *Enterobacter cloacae* A1137 (Identity: 95%), and *Klebsiella pneumoniae* KP486 (Identity: 94%) (Figure 3), indicating that ICE*Mmo*MP63 likely evolved from related ICEs, genomic islands, or plasmids of Enterobacteriaceae strains.

### ICE*Mmo*MP63 transfers into *E. coli* 25DN under polymyxin E pressure

**FIGURE 4:**
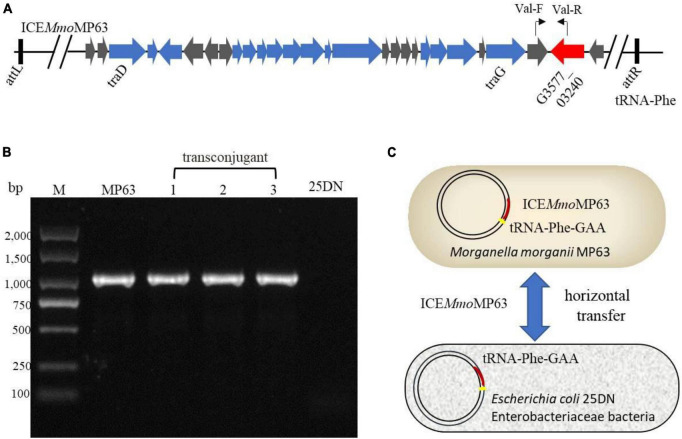
Verification of the transfer of ICE*Mmo*MP63. **(A)** Primer positions in ICE*Mmo*MP63 are indicated using bent arrows. **(B)** ICE*Mmo*MP63 fragment was amplified by PCR using total DNA from strain MP63, three transconjugants (lanes 1–3), and strain 25DN as templates. **(C)** Dissemination model of ICE*Mmo*MP63 with insertion site.

To determine whether ICE*Mmo*MP63 was an antibiotic-resistant ICE and could horizontally transfer among Enterobacteriaceae species, conjugation assays were conducted using MP63 with *E. coli* 25DN as the recipient strain. The transconjugation frequency was about 8.13 × 10^–7^ colony-forming units/donor with polymyxin E and sodium azide as the selective pressure. PCR testing and DNA sequencing results verified that a fragment of ICE*Mmo*MP63 was present in the transconjugants, and bioinformatic analysis revealed that both the *att* site (5′- GACACGGGGATTTTCAATCCC-3′) and insertion site (*tRNA-Phe* gene) of ICE*Mmo*MP63 were also present in the strain 25DN genome from position 929,323 to 929,343 bp (Figure 4), indicating that ICE*Mmo*MP63 was inserted into the *E. coli* 25DN genome. Further bioinformation analysis revealed that the integration site *tRNA-Phe* gene was conserved in many strains of Enterobacteriaceae, such as *E. coli* DL21 (GenBank accession no. CP079747), *K. pneumoniae* KPH3 (GenBank accession no. CP102552), *S. enterica* s15D023 (GenBank accession no. CP101340), *Serratia ureilytica* HNU47 (GenBank accession no. CP098030), *E. cloacae* complex sp. R_G8 (GenBank accession no. CP102246), and *Enterobacter asburiae* R_A5.MM (GenBank accession no. CP102247), suggesting that ICE*Mmo*MP63 might spread ARGs among Enterobacteriaceae. Additionally, MIC analysis showed that the transconjugant 25DN-MP acquired resistance to polymyxin E, tetracycline, and cefixime (Supplementary Table 2). The above results revealed that ICE*Mmo*MP63 was an antibiotic-resistant ICE that could horizontally transfer.

### The MFS transporter in ICE*Mmo*MP63 mediates multiple antibiotic resistance

Phylogenetic relationships analysis showed that the MFS transporter encoded by G3577_03020 is closely related to MFS transporters from other Enterobacteriaceae bacteria (Supplementary Figure 3). To determine whether the MFS transporter (G3577_03020) is responsible for the antibiotic resistance of ICE*Mmo*MP63, gene G3577_03020 was expressed in *E. coli* DH5α, and the recombinant strain was named M3020. MIC analysis showed that M3020 acquired resistance to cefixime, polymyxin E, and tetracycline (Supplementary Table 2), indicating that G3577_03020 mediates multiple antibiotic resistances.

The effects of the known EPIs CCCP, RES, VER, and NMP on G3577_03020 were then investigated. At a subinhibitory concentration (8 mg/L) of VER, a calcium antagonist, the MIC of strain M3020 was reduced from > 128 to 8 mg/L for cefixime, from 128 to 2 mg/L for polymyxin E, and from 64 to 16 mg/L for tetracycline (Table 1), while CCCP, NMP, and RES had no effect on the MICs. However, the effects of VER suggested that G3577_03020 can function as an efflux pump.

**TABLE 1:** Effects of EPIs on antibiotic MICs of strain M3020.

MICs^a^	No EPI	CCCP	NMP	VER	RES
Cefixime	> 128	128	128	8	128
Polymyxin E	128	128	128	2	128
Tetracycline	64	64	64	16	64

^a^MICs: mg/L. CCCP, cyanide m-chlorophenylhydrazone; EPI, efflux pump inhibitor; MICs, minimum inhibitory concentrations; NMP, N-methyl-2-pyrrolidone; RES, reserpine; VER, verapamil.

### Glabridin inhibits the growth of strain M3020

We further investigated whether novel EPIs could inhibit G3577_03020. Glabridin, an hydroxyisoflavan (Supplementary Figure 4), was identified as a potential G3577_03020 inhibitor following screening of a library of traditional Chinese medicine compounds. As shown in Table 2, in the presence of glabridin (especially with 25 μM glabridin), the MIC of strain M3020 was reduced from > 128 to 2 mg/L for cefixime, from 128 to 2 mg/L for polymyxin E, and from 64 to 8 mg/L for tetracycline, indicating glabridin could effectively reduce the MICs of tetracycline, polymyxin E, and cefixime for strain M3020.

**TABLE 2:** Effects of glabridin on the antibiotic MICs of strain M3020.

MICs^a^	No EPI	5 μM glabridin	15 μM glabridin	25 μM glabridin
Cefixime	>128	16	4	2
Polymyxin E	128	16	8	2
Tetracycline	64	16	16	8

^a^MICs: mg/L. MICs, minimum inhibitory concentrations.

### G3577_03020 binds to antibiotics and EPIs

**FIGURE 5:**
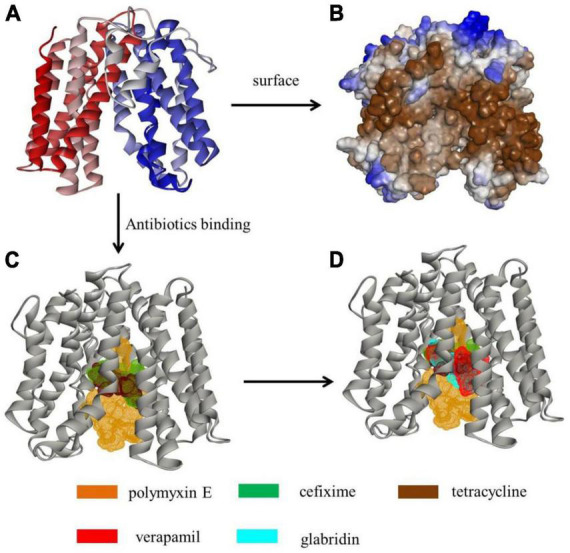
Tertiary structure modeling of MFS transporter G3577_03020 with antibiotics and EPIs. **(A)** Tertiary structure of G3577_03020. **(B)** Hydrophobicity of G3577_03020 surface and tunnel. **(C,D)** Molecular docking models showing embedding of **(C)** polymyxin E, cefixime, and tetracycline in the G3577_03020 cavity and **(D)** verapamil and glabridin in the G3577_03020 cavity.

As the inhibitory levels of tetracycline, polymyxin E, and cefixime for M3020 decreased in the presence of EPIs (VER and glabridin), molecular docking analysis was performed to determine whether G3577_03020 could bind to these antibiotics and EPIs. A homologous model of G3577_03020 was constructed based on the crystal structure of 7d5p.1.A, an efflux transporter from *Staphylococcus aureus*. In this model, G3577_03020 contained 12 transmembrane (TM) helices (TMs 1-12) and formed two domains (C domain and N domain), connected by a 44-residue linker (residues 205-248) (Figure 5A). Additionally, there was a central cavity in the TM core between the N and C domains and hydrophobic surface features (Figure 5B). Tetracycline, polymyxin E, and cefixime binding sites were located at the central cavity in the TM core (Figure 5C). Thr^124^, Trp^151^, Trp^124^, Leu^63^, Ala^155^, and Val^265^ of G3577_03020 were the main contributors for antibiotic binding (Supplementary Figures 5–7). Moreover, G3577_03020 showed binding with VER and glabridin, and interestingly, the EPI binding sites and antibiotic binding sites overlapped (Figure 5D), indicating that inhibition by VER and glabridin may be caused by competition for binding. Additional analysis revealed that amino acids involved in antibiotic binding, such as Leu^63^, Trp^151^, and Ala^155^, were occupied by VER and glabridin (Supplementary Figures 8, 9), further suggesting competitive binding by the EPIs.

## Discussion

Integrative and conjugative elements (ICEs) may be the major mechanism for horizontal transmission of ARGs among bacteria (Guglielmini et al., 2011; Fu et al., 2021). We identified an antibiotic-resistant ICE, named ICE*Mmo*MP63, in a MAR *M. morganii* MP63 strain isolated from hospital sewage in China. Conjugation assays showed that ICE*Mmo*MP63 could be horizontally transferred from MP63 to *E. coli* 25DN, and bioinformatics analysis revealed that ICE*Mmo*MP63 was integrated into the *E. coli* 25DN genome. Further bioinformation analysis revealed that the integration site *tRNA-Phe* was conserved in many strains of Enterobacteriaceae, suggesting that ICE*Mmo*MP63 might spread ARGs among Enterobacteriaceae. Phylogenetic analysis revealed that ICE*Mmo*MP63 was closely related to mobile elements in Enterobacterales species (Supplementary Figure 2), further suggesting the possibility of horizontal transmission.

Further analysis demonstrated that ICE*Mmo*MP63 encodes the MFS transporter (G3577_03020), a potential efflux pump responsible for the antibiotic resistance of ICE*Mmo*MP63. Bacterial efflux pumps, whereby bacteria pump out antibiotics, are major contributors to antibiotic resistance (Blair et al., 2015; Munita and Arias, 2016), and therefore there is interest in EPIs as therapeutic agents (Lomovskaya et al., 2001; Lomovskaya and Bostian, 2006; Lynch, 2006; Mahamoud et al., 2006; Stavri et al., 2007; Martins et al., 2008). By screening a traditional Chinese medicine library, we identified glabridin as a potential inhibitor of G3577_03020 and determined that glabridin reduced the MICs of several antibiotics. Further analyses suggested that glabridin competes with these antibiotics by binding to specific amino acids in G3577_03020. Our findings indicate that glabridin has potential for treating MAR bacterial infections and also lays the foundation for the design and optimization of glabridin as a new antimicrobial drug.

## Data availability statement

The datasets presented in this study can be found in online repositories. The names of the repository/repositories and accession number(s) can be found in the article/Supplementary material.

## Author contributions

JF: conceptualization and writing—original draft. YL and ZW: methodology and investigation. FW: resources. GZ: software and validation. CZ and GC: writing—review and editing, and supervision. All authors gave final approval for publication and agreed to be held accountable for the work performed therein.

## Footnotes

1
https://blast.ncbi.nlm.nih.gov/blast.cgi

2
https://ncbi.nlm.nih.gov/genome/annotation_prok/

3
https://www.uniprot.org/blast/
