# Supplementary material for: Glabridin inhibited the spread of polymyxin-resistant Enterobacterium carrying ICEMmoMP63
Source: Front Microbiol. 2023 May 22;14:1188900. doi: 10.3389/fmicb.2023.1188900 (PMC10239875; doi:10.3389/fmicb.2023.1188900)
Supplement: Supplementary file 1 [file Data_Sheet_1.docx]

**Supplementary Materials**

**Glabridin inhibited the spread of polymyxin-resistant Enterobacterium carrying ICE*Mmo*MP63**

Jiafang Fu ^1, 2^, Yayu Liu ^1, 2^, Fengtian Wang ^3^, Gongli Zong ^1, 2^, Zhen Wang ^4^, Chuanqing Zhong ^4*^, Guangxiang Cao ^1, 2*^

^1^ First Affiliated Hospital of Shandong First Medical University, Biomedical Sciences College & Shandong Medicinal Biotechnology Centre, Shandong First Medical University & Shandong Academy of Medical Sciences, Ji’nan, China

^2^ NHC Key Laboratory of Biotechnology Drugs (Shandong Academy of Medical Sciences), Ji’nan, China

^3^ Jinan Municipal Minzu Hospital, Ji’nan, China

^4^ School of Municipal and Environmental Engineering, Shandong Jianzhu University, Ji’nan, China

* Corresponding author:

Chuanqing Zhong

E-mail address: zhongchuanqing@sdjz.edu.cn (C. Zhong).

Guangxiang Cao

E-mail address: caoguangxiang@sdfmu.edu.cn (G. Cao).

**Running title:** Glabridin inhibits polymyxin transporter

Table S1 Primers used in this study.

| Name | DNA sequence(5'-3') |
| --- | --- |
| 27F | AGAGTTTGATCCTGGCTCAG |
| 1492R | GGTTACCTTGTTACGACTT |
| M3020-F | AATATTGAAAAAGGAAGAGTATGTCAAATACCATTTCTGT |
| M3020-R | TTAAAGGCTGTGTCGTTCCG |
| AP-F | GCCTCGTGATACGCCTATTT |
| AP-R | ACTCTTCCTTTTTCAATATT |
| Val-F | GCAGGGAAGTGGAGAAACTG |
| Val-R | GCCGCTGCTTACCTGACTGG |

**Table S2** Antibiotic susceptibility test of strains (MIC: mg/L)

| Strain | MP63 | 25DN-MP | 25DN | M3020 | DH5α |
| --- | --- | --- | --- | --- | --- |
| Cefixime | >128^R^ | >128^R^ | <2^S^ | >128^R^ | <2^S^ |
| Meropenem | 32^R^ | <2^S^ | <2^S^ | <2 ^S^ | <2^S^ |
| Polymyxin E | >128^R^ | 128^R^ | <2^S^ | 128^R^ | <2^S^ |
| Kanamycin | 16^S^ | 2^S^ | <2^S^ | 4 ^S^ | 4^S^ |
| Florfenicol | 16^R^ | 2^S^ | <2^S^ | 4 ^S^ | 4^S^ |
| Ciprofloxacin | <2^S^ | <2^S^ | <2^S^ | <2 ^S^ | <2^S^ |
| Tetracycline | 96^R^ | 64^R^ | 4^S^ | 64^R^ | 4^S^ |

“R” indicates resistant; “S” indicates susceptible. MIC breakpoint was based on CLSI (2017).

*Morganella morganii* MP63, *Escherichia coli* 25DN-MP, *E. coli* M3020, *E. coli* 25DN, and *E. coli* DH5α.

**Table S3 Genomic characteristics of strain MP63**

| Type | Name | Size (bp) | GC% | Protein | rRNA | tRNA | Other RNA | Gene | Pseudogene |
| --- | --- | --- | --- | --- | --- | --- | --- | --- | --- |
| Chromosome | - | 4004672 | 51.1 | 3,654 | 22 | 81 | 4 | 3,799 | 38 |
| Plasmid | pMP63A | 4982 | 36.9 | 4 | - | - | - | 4 | - |
| Plasmid | pMP63B | 2683 | 41.8 | 2 | - | - | - | 2 | - |
| Plasmid | pMP63C | 1741 | 24.8 | 1 | - | - | - | 2 | 1 |

**Table S4 Antibiotic resistance genes annotated in strain MP63.**

| **gene locus** | **Start** | **End** | **Stand** | **gene** | **Description** |
| --- | --- | --- | --- | --- | --- |
| Chromosome | 3582 | 5996 | + | *gyrB* | fluoroquinolone resistant gyrB |
| Chromosome | 94188 | 96641 | - | *pbp1a* | The enzyme has a penicillin-insensitive transglycosylase N-terminal domain (formation of linear glycan strands) and a penicillin-sensitive transpeptidase C-terminal domain (cross-linking of the peptide subunits) |
| Chromosome | 216262 | 217446 | + | *EF-Tu* | antibiotic target alteration |
| Chromosome | 355661 | 356509 | + | *sul4* | Putative sulfate transporter |
| Chromosome | 402095 | 403318 | - | *ampH* | Beta-lactamase |
| Chromosome | 481630 | 482814 | + | *mdtL* | Major facilitator superfamily transporter. Multidrug resistance efflux pump. |
| Chromosome | 783376 | 784791 | - | *lmrB* | ABC transporter system, Macrolide-Lincosamide-Streptogramin B efflux pump. |
| Chromosome | 871176 | 872051 | - | *blaSFO-1* | Beta-lactamase |
| Chromosome | 872162 | 873301 | + | *dha-1* | Class C beta-lactamase. This enzyme breaks the beta-lactam antibiotic ring open and deactivates the molecule's antibacterial properites. |
| Chromosome | 894124 | 894978 | + | *bacA* | Undecaprenyl pyrophosphate phosphatase, which consists in the sequestration of Undecaprenyl pyrophosphate. |
| Chromosome | 964930 | 968106 | - | *acrB* | Resistance-nodulation-cell division transporter system. Multidrug resistance efflux pump. |
| Chromosome | 968121 | 969218 | - | *acrA* | Resistance-nodulation-cell division transporter system. Multidrug resistance efflux pump. |
| Chromosome | 985730 | 987430 | - | *rosB* | Efflux pump/potassium antiporter system RosB: Potassium antiporter. |
| Chromosome | 1101546 | 1103039 | + | *dha2* | major facilitator superfamily (MFS) antibiotic efflux pump |
| Chromosome | 1156762 | 1158555 | + | *ftsI* | Penicillin-binding protein mutations conferring resistance to beta-lactam antibiotics |
| Chromosome | 1206753 | 1206872 | - | *bla* | class A Bacillus anthracis Bla beta-lactamase |
| Chromosome | 1321590 | 1322231 | + | *catA2* | Group A chloramphenicol acetyltransferase, which can inactivate chloramphenicol. |
| Chromosome | 1479043 | 1480221 | + | *bcr* | multidrug efflux pump Bcr |
| Chromosome | 1716447 | 1717097 | + | *catB3* | Group B chloramphenicol acetyltransferase, which can inactivate chloramphenicol. Also referred to as xenobiotic acetyltransferase. |
| Chromosome | 1820222 | 1822207 | + | *arnA* | Bifunctional enzyme that catalyzes the oxidative decarboxylation of UDP-glucuronic acid (UDP-GlcUA) to UDP-4-keto-arabinose (UDP-Ara4O) and the addition of a formyl group to UDP-4-amino-4-deoxy-L-arabinose (UDP-L-Ara4N) to form UDP-L-4-formamido-arabinose (UDP-L-Ara4FN). The modified arabinose is attached to lipid A and is required for resistance to polymyxin and cationic antimicrobial peptides. |
| Chromosome | 1855087 | 1856460 | - | *mdtK* | Major facilitator superfamily transporter. Multidrug resistance efflux pump. |
| Chromosome | 1905777 | 1906109 | + | *emrE* | Multidrug resistance efflux pump. |
| Chromosome | 2077061 | 2077423 | + | *aadB* | aminoglycoside nucleotidyltransferase ANT(2'')-Ia |
| Chromosome | 2080626 | 2080946 | + | *emrE* | Multidrug resistance efflux pump. |
| Chromosome | 2221139 | 2222338 | + | *mdtH* | Major facilitator superfamily transporter. Multidrug resistance efflux pump. |
| Chromosome | 2517995 | 2519227 | + | *mdtG* | Major facilitator superfamily transporter. Multidrug resistance efflux pump. |
| Chromosome | 2658364 | 2658795 | + | *FosA5* | Glutathione transferase, metalloglutathione transferase which confers resistance to fosfomycin by catalyzing the addition of glutathione to fosfomycin |
| Chromosome | 2776063 | 2778009 | - | *macB* | Resistance-nodulation-cell division transporter system. Multidrug resistance efflux pump. Macrolide-specific efflux system. |
| Chromosome | 2952791 | 2954722 | + | *pbpB* | The enzyme has a penicillin-insensitive transglycosylase N-terminal domain (formation of linear glycan strands) and a penicillin-sensitive transpeptidase C-terminal domain (cross-linking of the peptide subunits) |
| Chromosome | 2975693 | 2977225 | + | *EmrB* | major facilitator superfamily (MFS) antibiotic efflux pump |
| Chromosome | 3029594 | 3030013 | - | *tet(34)* | Xanthine-guanine phosphoribosyltransferase. Mechanism detail unknown. |
| Chromosome | 3231281 | 3233788 | - | *pbp1b* | The enzyme has a penicillin-insensitive transglycosylase N-terminal domain (formation of linear glycan strands) and a penicillin-sensitive transpeptidase C-terminal domain (cross-linking of the peptide subunits) |
| Chromosome | 3518741 | 3519553 | + | *ksgA* | Specifically dimethylates two adjacent adenosines in the loop of a conserved hairpin near the 3'-end of 16S rRNA in the 30S particle. Its inactivation leads to kasugamycin resistance. |
| Chromosome | 3520878 | 3521324 | - | *dfrA3* | Group A drug-insensitive dihydrofolate reductase, which can not be inhibited by trimethoprim. |
| Chromosome | 3532131 | 3533495 | + | *tolc* | Resistance-nodulation-cell division transporter system. Multidrug resistance efflux pump. |
| Chromosome | 3800146 | 3801330 | - | *EF-Tu* | elfamycin antibiotic |
| Chromosome | 3808035 | 3808160 | - | NA | resistance-nodulation-cell division (RND) antibiotic efflux pump |
| Chromosome | 3820784 | 3821371 | + | NA | resistance-nodulation-cell division (RND) antibiotic efflux pump |
| Chromosome | 3918926 | 3920113 | - | *emrd* | Multidrug resistance efflux pump. |
| pMP63A | 1764 | 3146 | + | *tetL* | Major facilitator superfamily transporter, tetracycline efflux pump. |
| pMP63B | 307 | 951 | + | *qnrD1* | Pentapeptide repeat family, which protects DNA gyrase from the inhibition of quinolones. |
| pMP63C | 59 | 1426 | - | *aph(2'')-Ia* | Aminoglycoside N-acetyltransferase, which modifies aminoglycosides by acetylation. |

**Table S5 Putative virulence factor-encoding genes annotated in strain MP63**

| Start | End | Stand | VFDB:Identity | VFDB:VF_id | VFDB:Type | VFDB: gene |
| --- | --- | --- | --- | --- | --- | --- |
| 18041 | 19024 | - | 42.4 | VFG043092 | Predicted | *tar/cheM* |
| 21413 | 22765 | - | 52 | VFG047258 | Predicted | *FN3523_0439* |
| 38657 | 39232 | - | 41.2 | VFG043577 | Predicted | *gbpA* |
| 54243 | 56177 | - | 45.7 | VFG043040 | Predicted | *tar/cheM* |
| 68852 | 69625 | + | 43.2 | VFG048480 | Predicted | *A225_1601* |
| 98735 | 99790 | + | 40.4 | VFG013123 | Predicted | *comE/pilQ* |
| 103996 | 104670 | + | 63.1 | VFG046612 | Predicted | *FN3523_1292* |
| 113571 | 114191 | - | 42.9 | VFG001867 | Verified | *sodB* |
| 140916 | 142301 | + | 42.8 | VFG019760 | Predicted | *pilR* |
| 146991 | 147323 | + | 43.9 | VFG042643 | Predicted | *mrxJ* |
| 147482 | 150511 | - | 44.3 | VFG035975 | Predicted | *ehaA* |
| 154444 | 156090 | + | 45.9 | VFG038764 | Predicted | *flrA* |
| 160326 | 160637 | + | 45.7 | VFG042629 | Predicted | *mrpJ* |
| 175246 | 177453 | - | 45.3 | VFG047394 | Predicted | *feoB* |
| 177649 | 177876 | - | 50 | VFG045722 | Predicted | *feoA* |
| 181254 | 181973 | + | 41.8 | VFG038219 | Verified | *bfmR* |
| 216262 | 217446 | + | 82.2 | VFG046459 | Predicted | *Fphi_1039* |
| 241483 | 242025 | - | 40.3 | VFG045340 | Verified | *ricA* |
| 268104 | 269096 | + | 54 | VFG013510 | Predicted | *wecA* |
| 269125 | 270180 | + | 65.7 | VFG023778 | Predicted | *YE105_C0173* |
| 270289 | 271419 | + | 73.1 | VFG007640 | Predicted | *wbjD/wecB* |
| 271416 | 272678 | + | 67.9 | VFG014138 | Predicted | *orfH* |
| 272675 | 273748 | + | 79.8 | VFG007659 | Predicted | *rmlB* |
| 273758 | 274639 | + | 77.6 | VFG048826 | Predicted | *KPHS_35570* |
| 284906 | 285643 | - | 44.1 | VFG013199 | Predicted | *hemD* |
| 285640 | 286725 | - | 62.7 | VFG013198 | Predicted | *hemC* |
| 329980 | 330576 | + | 45.4 | VFG001864 | Verified | *mip* |
| 332023 | 333393 | - | 59.3 | VFG038902 | Predicted | *hlyA* |
| 356502 | 357836 | + | 75.2 | VFG013514 | Predicted | *mrsA/glmM* |
| 358920 | 359372 | + | 51.7 | VFG039487 | Predicted | *CbuG_0575* |
| 367987 | 368874 | + | 73.2 | VFG043545 | Predicted | *ECS88_3547* |
| 387996 | 388931 | - | 42.7 | VFG047692 | Predicted | *FN3523_0020* |
| 389199 | 390251 | - | 41.8 | VFG015903 | Predicted | *argK* |
| 411668 | 411934 | - | 59.5 | VFG014114 | Predicted | *PA3144* |
| 417480 | 417749 | - | 41.7 | VFG042643 | Predicted | *mrxJ* |
| 425104 | 427317 | + | 63.1 | VFG048614 | Predicted | *iutA* |
| 436376 | 437110 | - | 49.2 | VFG042634 | Predicted | *mrfD* |
| 437779 | 439368 | - | 47.1 | VFG042647 | Predicted | *mrxC* |
| 455641 | 457167 | - | 57.4 | VFG009612 | Predicted | *narH* |
| 457197 | 460958 | - | 48 | VFG009597 | Predicted | *narG* |
| 466615 | 466908 | + | 46.7 | VFG042629 | Predicted | *mrpJ* |
| 478500 | 478808 | + | 42 | VFG042629 | Predicted | *mrpJ* |
| 486450 | 487565 | + | 51.7 | VFG030695 | Predicted | *sugC* |
| 491774 | 492088 | + | 45.1 | VFG042643 | Predicted | *mrxJ* |
| 493710 | 494447 | - | 40.4 | VFG003532 | Predicted | *ysaJ* |
| 499651 | 501711 | + | 51.2 | VFG003525 | Predicted | *ysaV* |
| 502147 | 503439 | + | 53.7 | VFG000555 | Verified | *invC* |
| 505483 | 506145 | + | 56.4 | VFG003721 | Predicted | *spaP* |
| 506147 | 506413 | + | 54 | VFG003518 | Predicted | *ysaS* |
| 507174 | 508223 | + | 42.5 | VFG041463 | Predicted | *spaS1* |
| 508332 | 508817 | + | 41.5 | VFG041462 | Predicted | *spaT1* |
| 514406 | 515209 | + | 45.9 | VFG045346 | Verified | *IlpA* |
| 525614 | 526627 | - | 40.4 | VFG044101 | Predicted | *phuU* |
| 539543 | 540679 | - | 76.6 | VFG044234 | Predicted | *pmsA* |
| 553086 | 553784 | - | 44.4 | VFG001145 | Verified | *hifB* |
| 571551 | 572696 | + | 58.1 | VFG043568 | Predicted | *ompD* |
| 574253 | 574816 | - | 74.9 | VFG042620 | Predicted | *mrpI* |
| 575470 | 575997 | + | 92.6 | VFG042621 | Predicted | *mrpA* |
| 576109 | 576624 | + | 54.8 | VFG042622 | Predicted | *mrpB* |
| 576646 | 579303 | + | 71.2 | VFG042623 | Predicted | *mrpC* |
| 579300 | 580058 | + | 72.2 | VFG042624 | Predicted | *mrpD* |
| 580077 | 580613 | + | 57.9 | VFG042625 | Predicted | *mrpE* |
| 580625 | 581107 | + | 73.2 | VFG042626 | Predicted | *mrpF* |
| 581118 | 581663 | + | 69.2 | VFG042627 | Predicted | *mrpG* |
| 581683 | 582501 | + | 65.9 | VFG042628 | Predicted | *mrpH* |
| 582568 | 582927 | + | 55.7 | VFG042629 | Predicted | *mrpJ* |
| 582908 | 583468 | + | 57.1 | VFG048231 | Predicted | *Kvar_0780* |
| 583989 | 584573 | + | 41.8 | VFG042631 | Predicted | *mrfA* |
| 585151 | 587784 | + | 55.5 | VFG042633 | Predicted | *mrfC* |
| 587781 | 588539 | + | 58 | VFG042634 | Predicted | *mrfD* |
| 588590 | 589090 | + | 41.1 | VFG042638 | Predicted | *mrfH* |
| 591209 | 591760 | - | 56.8 | VFG048223 | Predicted | *KOX_22330* |
| 592092 | 592460 | + | 47.3 | VFG042629 | Predicted | *mrpJ* |
| 600734 | 603352 | + | 40.3 | VFG021094 | Predicted | *misL* |
| 617216 | 617782 | + | 47.5 | VFG002308 | Verified | *pilL* |
| 683009 | 684208 | + | 43.8 | VFG036945 | Predicted | *farA* |
| 684212 | 685744 | + | 43.6 | VFG036966 | Predicted | *farB* |
| 698523 | 700169 | + | 73.8 | VFG045692 | Predicted | *htpB* |
| 731507 | 733018 | - | 43.6 | VFG019760 | Predicted | *pilR* |
| 775247 | 775930 | + | 40.9 | VFG031738 | Predicted | *regX3* |
| 777493 | 779061 | - | 45.9 | VFG025878 | Predicted | *tsr* |
| 803814 | 804362 | - | 40.9 | VFG045476 | Predicted | *lpg0021* |
| 812295 | 813731 | - | 55.7 | VFG042736 | Predicted | *rpoN* |
| 816200 | 817168 | - | 46.6 | VFG011729 | Predicted | *kpsF* |
| 823437 | 824525 | - | 41.6 | VFG014995 | Predicted | *algW* |
| 832147 | 833541 | - | 45.8 | VFG014995 | Predicted | *algW* |
| 849418 | 850008 | - | 41.4 | VFG013423 | Predicted | *gmhA/lpcA* |
| 887290 | 889140 | - | 53.1 | VFG009718 | Predicted | *sigA/rpoV* |
| 914052 | 914606 | - | 43.9 | VFG009680 | Predicted | *ahpC* |
| 945885 | 946673 | + | 65.3 | VFG000077 | Verified | *clpP* |
| 953288 | 953620 | + | 43.8 | VFG043478 | Predicted | *comE1* |
| 964930 | 968106 | - | 74.5 | VFG049139 | Predicted | *acrB* |
| 968121 | 969218 | - | 64.6 | VFG049129 | Predicted | *KPHS_11890* |
| 981509 | 982465 | + | 48.4 | VFG013617 | Predicted | *hemH* |
| 990071 | 992950 | + | 56.9 | VFG015944 | Predicted | *PSEEN2697* |
| 993381 | 996179 | + | 57 | VFG015944 | Predicted | *PSEEN2697* |
| 996635 | 999496 | + | 56.6 | VFG015944 | Predicted | *PSEEN2697* |
| 1001036 | 1003813 | - | 42.9 | VFG031407 | Predicted | *ctpV* |
| 1004516 | 1006204 | + | 51.5 | VFG042343 | Predicted | *ETAE_0910* |
| 1006241 | 1010875 | + | 40 | VFG042385 | Predicted | *shlA* |
| 1023431 | 1024153 | - | 52.2 | VFG013325 | Predicted | *lpxH* |
| 1100313 | 1101503 | + | 44.2 | VFG036940 | Predicted | *farA* |
| 1101546 | 1103039 | + | 51.7 | VFG036964 | Predicted | *farB* |
| 1135292 | 1136491 | - | 54.7 | VFG039278 | Predicted | *CbuG_1738* |
| 1142226 | 1142828 | - | 45.5 | VFG009376 | Predicted | *leuD* |
| 1171880 | 1172797 | + | 74 | VFG013414 | Predicted | *lpxC* |
| 1180204 | 1181727 | - | 48.3 | VFG045991 | Predicted | *hofB* |
| 1181753 | 1182172 | - | 47.8 | VFG045972 | Predicted | *ppdD* |
| 1234837 | 1235646 | - | 57.8 | VFG041818 | Predicted | *plu3775* |
| 1237842 | 1238465 | + | 42 | VFG013192 | Predicted | *hitC* |
| 1256067 | 1257083 | + | 60.4 | VFG002361 | Verified | *galE* |
| 1262239 | 1264278 | + | 65 | VFG012633 | Predicted | *ireA* |
| 1353670 | 1354329 | + | 40 | VFG015785 | Predicted | *gacA* |
| 1471367 | 1471723 | - | 40.9 | VFG042629 | Predicted | *mrpJ* |
| 1494048 | 1495145 | + | 76.2 | VFG044280 | Predicted | *PMI0229* |
| 1495147 | 1495965 | + | 67.5 | VFG044281 | Predicted | *PMI0230* |
| 1496146 | 1498206 | + | 45.3 | VFG007263 | Predicted | *irgA* |
| 1514966 | 1515721 | + | 78.7 | VFG038840 | Predicted | *flmH* |
| 1515877 | 1516113 | + | 63.2 | VFG011430 | Verified | *acpXL* |
| 1516218 | 1517426 | + | 41.7 | VFG009135 | Predicted | *kasB* |
| 1534285 | 1534923 | + | 40 | VFG015384 | Predicted | *Pmen_2312* |
| 1544704 | 1546173 | - | 55.5 | VFG000474 | Predicted | *phoQ* |
| 1546206 | 1546880 | - | 71.9 | VFG004061 | Predicted | *phoP* |
| 1569452 | 1570279 | - | 55.7 | VFG045346 | Verified | *IlpA* |
| 1709283 | 1710815 | - | 68.2 | VFG037041 | Predicted | *katA* |
| 1716447 | 1717097 | + | 40.7 | VFG005041 | Predicted | *cap5H* |
| 1723682 | 1724287 | - | 48.4 | VFG011225 | Predicted | *pagP* |
| 1752604 | 1753644 | - | 44.2 | VFG047558 | Predicted | *Fphi_0419* |
| 1802027 | 1802881 | - | 55.6 | VFG034233 | Predicted | *sitD* |
| 1802878 | 1803753 | - | 67.1 | VFG012585 | Predicted | *sitC* |
| 1803750 | 1804634 | - | 64.3 | VFG034214 | Predicted | *sitB* |
| 1804631 | 1805632 | - | 66.2 | VFG012575 | Predicted | *sitA* |
| 1816160 | 1817170 | + | 43.6 | VFG044101 | Predicted | *phuU* |
| 1818087 | 1819232 | + | 41.5 | VFG011158 | Predicted | *bplF* |
| 1829135 | 1829662 | + | 61.3 | VFG000463 | Verified | *sodCI* |
| 1830948 | 1831748 | - | 44.5 | VFG044172 | Verified | *chuV* |
| 1831750 | 1832742 | - | 55.7 | VFG044136 | Predicted | *hmuU* |
| 1832739 | 1833569 | - | 56 | VFG044137 | Predicted | *hmuT* |
| 1833566 | 1834633 | - | 55.2 | VFG044292 | Predicted | *hmuS* |
| 1835129 | 1836178 | - | 52.3 | VFG044185 | Predicted | *VVA1298* |
| 1862572 | 1863150 | - | 67 | VFG001867 | Verified | *sodB* |
| 1883867 | 1884868 | - | 41 | VFG013992 | Predicted | *pilR* |
| 1889411 | 1890946 | + | 42.8 | VFG043148 | Predicted | *lafK* |
| 1895781 | 1896122 | - | 40.3 | VFG042629 | Predicted | *mrpJ* |
| 1918386 | 1919381 | - | 50 | VFG005359 | Predicted | *plr/gapA* |
| 1933065 | 1933970 | + | 69.3 | VFG013348 | Predicted | *galU* |
| 1933993 | 1935336 | + | 70.9 | VFG037990 | Predicted | *BJAB07104_00096* |
| 1935345 | 1936358 | + | 68.5 | VFG049098 | Predicted | *A225_3875* |
| 1938338 | 1941055 | - | 49.3 | VFG006719 | Predicted | *lap* |
| 1966406 | 1967077 | - | 43.2 | VFG009863 | Predicted | *mprA* |
| 2027454 | 2029067 | + | 41.1 | VFG038764 | Predicted | *flrA* |
| 2034101 | 2034853 | + | 46.7 | VFG018301 | Predicted | *spvC* |
| 2112701 | 2115397 | - | 68 | VFG018402 | Predicted | *mgtB* |
| 2118990 | 2119601 | + | 41.7 | VFG033805 | Verified | *nleD* |
| 2156759 | 2158300 | + | 42.1 | VFG019760 | Predicted | *pilR* |
| 2162393 | 2163412 | + | 41.3 | VFG013711 | Predicted | *hmuU* |
| 2191596 | 2192264 | - | 40.5 | VFG006826 | Predicted | *lisR* |
| 2198021 | 2199001 | - | 57.3 | VFG013087 | Predicted | *msbB2* |
| 2206528 | 2207115 | - | 55 | VFG039536 | Verified | *CBU_1566* |
| 2230135 | 2230986 | - | 78 | VFG013470 | Predicted | *kdsA* |
| 2233811 | 2235136 | - | 52 | VFG013196 | Predicted | *hemA* |
| 2271371 | 2272345 | + | 43.1 | VFG013197 | Predicted | *hemB* |
| 2383165 | 2383926 | - | 40.7 | VFG041321 | Verified | *lpnE* |
| 2401800 | 2402450 | + | 88.7 | VFG049010 | Predicted | *A225_4123* |
| 2437003 | 2437515 | - | 66.5 | VFG002318 | Verified | *fliZ* |
| 2437573 | 2438307 | - | 73.9 | VFG002319 | Verified | *fliA* |
| 2438619 | 2439689 | - | 57.5 | VFG011232 | Predicted | *flaA* |
| 2439920 | 2441071 | - | 57.2 | VFG011232 | Predicted | *flaA* |
| 2441437 | 2442846 | + | 40.5 | VFG002324 | Verified | *fliD* |
| 2442852 | 2443250 | + | 56.8 | VFG043053 | Predicted | *fliS* |
| 2447841 | 2448224 | - | 54.5 | VFG043106 | Predicted | *fliE* |
| 2448421 | 2450127 | + | 53.3 | VFG002657 | Predicted | *fliF* |
| 2450124 | 2451116 | + | 76.7 | VFG002658 | Predicted | *fliG* |
| 2451109 | 2451840 | + | 46.2 | VFG023639 | Predicted | *fliH* |
| 2451837 | 2453201 | + | 74.4 | VFG023641 | Predicted | *fliI* |
| 2453213 | 2453656 | + | 52.8 | VFG002661 | Predicted | *fliJ* |
| 2453656 | 2455023 | + | 43 | VFG002333 | Verified | *fliK* |
| 2455188 | 2455673 | + | 47.2 | VFG002663 | Predicted | *fliL* |
| 2455679 | 2456692 | + | 73.7 | VFG002335 | Verified | *fliM* |
| 2456712 | 2457095 | + | 68.2 | VFG003423 | Predicted | *fliN* |
| 2457097 | 2457564 | + | 44.8 | VFG002337 | Verified | *fliO* |
| 2457573 | 2458313 | + | 75.1 | VFG023655 | Predicted | *fliP* |
| 2458343 | 2458612 | + | 77.5 | VFG043118 | Predicted | *fliQ* |
| 2458616 | 2459401 | + | 60.8 | VFG002669 | Predicted | *fliR* |
| 2461438 | 2462385 | - | 42.9 | VFG002670 | Predicted | *flgL* |
| 2462410 | 2464050 | - | 42.8 | VFG018995 | Predicted | *flgK* |
| 2464201 | 2465160 | - | 52 | VFG002343 | Verified | *flgJ* |
| 2465178 | 2466284 | - | 73.6 | VFG003028 | Predicted | *flgI* |
| 2466302 | 2467051 | - | 70.8 | VFG043028 | Predicted | *flgH* |
| 2467128 | 2467910 | - | 76.2 | VFG023670 | Predicted | *flgG* |
| 2467930 | 2468685 | - | 57.4 | VFG002347 | Verified | *flgF* |
| 2468707 | 2469918 | - | 53.2 | VFG043077 | Predicted | *flgE* |
| 2470766 | 2471170 | - | 73.9 | VFG002350 | Verified | *flgC* |
| 2471176 | 2471592 | - | 60.1 | VFG043022 | Predicted | *flgB* |
| 2471717 | 2472424 | + | 44.2 | VFG023682 | Predicted | *flgA* |
| 2472539 | 2472859 | + | 47.1 | VFG002506 | Verified | *flgM* |
| 2485672 | 2487729 | - | 74.7 | VFG023690 | Predicted | *flhA* |
| 2487761 | 2488912 | - | 58.3 | VFG017341 | Predicted | *flhB* |
| 2489620 | 2490258 | - | 49.5 | VFG043205 | Verified | *cheZ* |
| 2490284 | 2490676 | - | 82.9 | VFG043206 | Verified | *cheY* |
| 2490718 | 2491785 | - | 61.3 | VFG025837 | Predicted | *cheB* |
| 2491757 | 2492527 | - | 59.2 | VFG043208 | Verified | *cheR* |
| 2492740 | 2494314 | - | 47.2 | VFG043209 | Verified | *cheD* |
| 2494427 | 2496112 | - | 48.5 | VFG043209 | Verified | *cheD* |
| 2496208 | 2496705 | - | 75.9 | VFG043093 | Predicted | *cheW* |
| 2496722 | 2498794 | - | 61.1 | VFG043042 | Predicted | *cheA* |
| 2498871 | 2499872 | - | 58.9 | VFG043212 | Verified | *motB* |
| 2499880 | 2500767 | - | 69.4 | VFG043213 | Verified | *motA* |
| 2500867 | 2501448 | - | 79.6 | VFG002358 | Verified | *flhC* |
| 2501454 | 2501645 | - | 63.5 | VFG043046 | Predicted | *flhD* |
| 2503520 | 2504215 | - | 60.7 | VFG004044 | Predicted | *mgtC* |
| 2517029 | 2517970 | + | 56.9 | VFG013438 | Predicted | *htrB* |
| 2529492 | 2530574 | - | 43.8 | VFG013192 | Predicted | *hitC* |
| 2548169 | 2548594 | - | 42.1 | VFG031462 | Predicted | *ndk* |
| 2558935 | 2560782 | - | 40.4 | VFG043573 | Predicted | *CT396* |
| 2572362 | 2573702 | - | 41.8 | VFG019760 | Predicted | *pilR* |
| 2619516 | 2620094 | - | 65.4 | VFG000121 | Verified | *algU* |
| 2671333 | 2672856 | - | 42.2 | VFG043040 | Predicted | *tar/cheM* |
| 2675367 | 2675603 | + | 45.9 | VFG042643 | Predicted | *mrxJ* |
| 2675749 | 2676297 | + | 59.8 | VFG042679 | Predicted | *fimA* |
| 2676500 | 2677198 | + | 54.5 | VFG042681 | Predicted | *fimC* |
| 2677242 | 2679830 | + | 52.2 | VFG042682 | Predicted | *fimD* |
| 2679838 | 2680839 | + | 57.6 | VFG042683 | Predicted | *fimH* |
| 2680851 | 2681384 | + | 43.8 | VFG042684 | Predicted | *fimF* |
| 2698287 | 2699360 | + | 67.1 | VFG043544 | Predicted | *Z1307* |
| 2721568 | 2722668 | + | 63.6 | VFG043568 | Predicted | *ompD* |
| 2738212 | 2738961 | - | 69.2 | VFG038845 | Predicted | *nueA* |
| 2739826 | 2740809 | - | 55.8 | VFG013242 | Verified | *lpxK* |
| 2740822 | 2742567 | - | 70.4 | VFG013253 | Predicted | *msbA* |
| 2772848 | 2775133 | - | 43.9 | VFG049193 | Predicted | *KPHS_39850* |
| 2804274 | 2804870 | + | 43 | VFG014106 | Predicted | *hisH2* |
| 2812243 | 2813616 | + | 87.1 | VFG048830 | Verified | *gnd* |
| 2834124 | 2834897 | + | 41.6 | VFG044378 | Predicted | *qbsC* |
| 2844743 | 2845435 | + | 42 | VFG048387 | Predicted | *KPN2242_19350* |
| 2867849 | 2868886 | - | 50.3 | VFG047511 | Predicted | *bioB* |
| 2883415 | 2884467 | - | 50.9 | VFG044185 | Predicted | *VVA1298* |
| 2921404 | 2921853 | + | 87.1 | VFG000478 | Predicted | *fur* |
| 2968450 | 2969655 | + | 42 | VFG030724 | Predicted | *sugC* |
| 2974502 | 2975680 | + | 45.6 | VFG036946 | Predicted | *farA* |
| 2975693 | 2977225 | + | 57.5 | VFG036972 | Predicted | *farB* |
| 2994648 | 2997128 | - | 81.5 | VFG049190 | Predicted | *KOX_00005* |
| 3005210 | 3006304 | - | 43.8 | VFG044185 | Predicted | *VVA1298* |
| 3012655 | 3013131 | - | 73.1 | VFG018243 | Predicted | *luxS* |
| 3016103 | 3016258 | - | 70.8 | VFG010906 | Predicted | *csrA* |
| 3044442 | 3045122 | - | 78.1 | VFG013421 | Predicted | *gmhA/lpcA* |
| 3055981 | 3056712 | + | 52.1 | VFG045607 | Verified | *lpg2936* |
| 3058762 | 3059886 | - | 48 | VFG013904 | Predicted | *pilT* |
| 3061146 | 3061742 | + | 56.9 | VFG013265 | Verified | *orfM* |
| 3061735 | 3062865 | + | 68.2 | VFG013626 | Predicted | *hemN* |
| 3071626 | 3073614 | - | 98.9 | VFG044296 | Predicted | *PMI2596* |
| 3073787 | 3075100 | + | 99.8 | VFG044297 | Predicted | *nrpX* |
| 3075890 | 3081994 | + | 99.2 | VFG044299 | Predicted | *nrpR* |
| 3082007 | 3091219 | + | 98.6 | VFG044300 | Predicted | *nrpS* |
| 3091219 | 3092307 | + | 99.2 | VFG044301 | Predicted | *nrpU* |
| 3092307 | 3093077 | + | 98 | VFG044302 | Predicted | *nrpT* |
| 3093100 | 3094866 | + | 98.5 | VFG044303 | Predicted | *nrpA* |
| 3094859 | 3096586 | + | 99.7 | VFG044304 | Predicted | *nrpB* |
| 3096779 | 3097348 | + | 98.9 | VFG044305 | Predicted | *nrpG* |
| 3098181 | 3098447 | + | 59.5 | VFG014114 | Predicted | *PA3144* |
| 3110503 | 3111114 | - | 46.7 | VFG036559 | Predicted | *fbpC* |
| 3112907 | 3113890 | - | 46.6 | VFG013190 | Predicted | *hitA* |
| 3170124 | 3171887 | + | 50.3 | VFG047605 | Predicted | *FNFX1_1207* |
| 3196843 | 3198141 | - | 58.7 | VFG005579 | Predicted | *eno* |
| 3200951 | 3203185 | - | 44.2 | VFG045730 | Predicted | *relA* |
| 3217969 | 3219249 | + | 70.4 | VFG013203 | Predicted | *hemL* |
| 3219307 | 3219543 | - | 42.1 | VFG041108 | Predicted | *sciR* |
| 3240945 | 3241832 | + | 40.1 | VFG022615 | Predicted | *panC* |
| 3244450 | 3246156 | - | 53.3 | VFG044153 | Predicted | *hasD* |
| 3246624 | 3248078 | - | 53.1 | VFG043807 | Predicted | *PMI0279* |
| 3260816 | 3261865 | + | 41 | VFG030684 | Predicted | *sugC* |
| 3296204 | 3298054 | - | 41.4 | VFG038763 | Verified | *fleQ/flrC* |
| 3309433 | 3310542 | - | 42.5 | VFG030314 | Predicted | *adhD* |
| 3412694 | 3413686 | - | 77.6 | VFG000477 | Predicted | *rpoS* |
| 3432509 | 3433090 | - | 44.1 | VFG002552 | Verified | *wcbN* |
| 3435001 | 3435816 | + | 65.7 | VFG045346 | Verified | *IlpA* |
| 3446634 | 3447788 | - | 64.5 | VFG013386 | Predicted | *lpxB* |
| 3447803 | 3448594 | - | 64.9 | VFG013394 | Predicted | *lpxA* |
| 3448598 | 3449050 | - | 44.8 | VFG011402 | Predicted | *fabZ* |
| 3449154 | 3450092 | - | 66 | VFG013379 | Predicted | *lpxD* |
| 3453235 | 3454590 | - | 46.3 | VFG015009 | Predicted | *mucP* |
| 3454617 | 3455477 | - | 41.1 | VFG045682 | Predicted | *EFAU004_01723* |
| 3455467 | 3456240 | - | 45.4 | VFG045688 | Predicted | *uppS* |
| 3532131 | 3533495 | + | 63.9 | VFG044140 | Predicted | *hasF* |
| 3538101 | 3539540 | - | 69.3 | VFG000331 | Verified | *rfaE* |
| 3551882 | 3555109 | - | 56.4 | VFG047713 | Predicted | *FNFX1_0026* |
| 3555124 | 3556263 | - | 49.1 | VFG047720 | Predicted | *Fphi_0805* |
| 3569138 | 3571060 | - | 58.8 | VFG043573 | Predicted | *CT396* |
| 3601385 | 3602020 | - | 57.7 | VFG006496 | Predicted | *ureG* |
| 3603505 | 3605223 | - | 57.5 | VFG019522 | Predicted | *ureB* |
| 3605291 | 3605713 | - | 52.5 | VFG006470 | Predicted | *ureA* |
| 3605743 | 3606045 | - | 48.5 | VFG000269 | Verified | *ureA* |
| 3633669 | 3634124 | - | 40.8 | VFG039382 | Predicted | *coxH2/rimL* |
| 3653307 | 3653819 | - | 53.8 | VFG013201 | Predicted | *hemG* |
| 3687156 | 3689732 | + | 43.4 | VFG015298 | Predicted | *hopL1* |
| 3690727 | 3692694 | + | 68.4 | VFG015389 | Predicted | *ppkA* |
| 3692694 | 3693419 | + | 57.5 | VFG015381 | Predicted | *PSPTO_2875* |
| 3693553 | 3694623 | + | 57.7 | VFG015375 | Predicted | *Psyr_2627* |
| 3694826 | 3696241 | + | 58.6 | VFG015368 | Predicted | *Psyr_2626* |
| 3736126 | 3736368 | + | 43.5 | VFG042629 | Predicted | *mrpJ* |
| 3736417 | 3736776 | + | 49.2 | VFG042643 | Predicted | *mrxJ* |
| 3743392 | 3745038 | - | 74.9 | VFG013186 | Predicted | *pgi* |
| 3767534 | 3768601 | - | 78.2 | VFG013612 | Predicted | *hemE* |
| 3773266 | 3774294 | + | 41.5 | VFG030695 | Predicted | *sugC* |
| 3775046 | 3776623 | - | 52.8 | VFG043040 | Predicted | *tar/cheM* |
| 3779731 | 3780489 | + | 43 | VFG044378 | Predicted | *qbsC* |
| 3800146 | 3801330 | - | 82.2 | VFG046459 | Predicted | *Fphi_1039* |
| 3820784 | 3821371 | + | 66.7 | VFG042734 | Predicted | *vfr* |
| 3844275 | 3845273 | - | 41.4 | VFG016532 | Predicted | *oppF* |
| 3845266 | 3846249 | - | 40.4 | VFG016532 | Predicted | *oppF* |
| 3876232 | 3877641 | + | 51.7 | VFG009407 | Predicted | *glnA1* |
| 3878863 | 3880317 | + | 40.3 | VFG019760 | Predicted | *pilR* |
| 3880314 | 3881684 | - | 70.1 | VFG013205 | Predicted | *hemN* |
| 3938812 | 3940209 | + | 57.8 | VFG002363 | Verified | *manC* |
| 3940212 | 3941576 | + | 68.9 | VFG019017 | Predicted | *YPK_3178* |
| 3941671 | 3943212 | + | 50.9 | VFG013399 | Predicted | *HD1598* |
| 3950592 | 3951530 | + | 78.1 | VFG000332 | Verified | *rfaD* |
| 3951541 | 3952602 | + | 61.3 | VFG013400 | Verified | *rfaF* |
| 3952587 | 3953564 | + | 58 | VFG013280 | Predicted | *opsX/rfaC* |
| 3957985 | 3959376 | + | 53 | VFG013311 | Predicted | *kdtA* |
| 3959376 | 3960155 | + | 49.6 | VFG013317 | Predicted | *lgtF* |
| 3960152 | 3960637 | + | 43.3 | VFG000320 | Verified | *kdtB* |

**Table S6 Genes annotated in ICE*Mmo*MP63**

| **locus_tag** | **start** | **stop** | **strand** | **length** | **gene** | **function** |
| --- | --- | --- | --- | --- | --- | --- |
| G3577_02795 | 604232 | 605107 | + | 876 | *parA* | chromosome partitioning protein ParA |
| G3577_02800 | 605100 | 606470 | + | 1371 | *dnaB* | replicative DNA helicase |
| G3577_02805 | 606467 | 608167 | + | 1701 | *parB* | chromosome partitioning protein ParB |
| G3577_02810 | 608160 | 608870 | + | 711 |  | DUF2786 domain-containing protein |
| G3577_02815 | 608877 | 609467 | + | 591 |  | DUF2857 domain-containing protein |
| G3577_02820 | 609464 | 609712 | + | 249 |  | hypothetical protein |
| G3577_02825 | 609812 | 611050 | + | 1239 |  | helix-turn-helix domain-containing protein |
| G3577_02830 | 611431 | 612144 | + | 714 |  | TIGR03761 family integrating conjugative element protein |
| G3577_02835 | 612146 | 612700 | + | 555 |  | hypothetical protein |
| G3577_02840 | 612716 | 614737 | + | 2022 |  | DNA topoisomerase III |
| G3577_02845 | 615375 | 615845 | + | 471 |  | DUF3577 domain-containing protein |
| G3577_02850 | 615906 | 616442 | + | 537 |  | single-stranded DNA-binding protein |
| G3577_02855 | 616507 | 616749 | + | 243 |  | DUF4160 domain-containing protein |
| G3577_02860 | 616733 | 616981 | + | 249 |  | DUF2442 domain-containing protein |
| G3577_02865 | 617117 | 617782 | + | 666 | *pilL* | pilus assembly protein PilL |
| G3577_02870 | 617779 | 618537 | + | 759 |  | hypothetical protein |
| G3577_02875 | 618549 | 619271 | + | 723 |  | TIGR03759 family integrating conjugative element protein |
| G3577_02880 | 619250 | 619885 | + | 636 |  | lytic transglycosylase domain-containing protein |
| G3577_02885 | 619891 | 620412 | + | 522 |  | integrating conjugative element protein |
| G3577_02890 | 620412 | 620984 | + | 573 |  | restriction endonuclease |
| G3577_02895 | 620995 | 621483 | + | 489 |  | hypothetical protein |
| G3577_02900 | 621476 | 623575 | + | 2100 | *traD* | type IV conjugative transfer system coupling protein TraD T4CP |
| G3577_02905 | 623568 | 624326 | + | 759 |  | TIGR03747 family integrating conjugative element membrane protein |
| G3577_02910 | 624591 | 625601 | - | 1011 | *IS110* | IS110 family transposase |
| G3577_02915 | 625708 | 626676 | - | 969 |  | pyridoxal-phosphate dependent enzyme |
| G3577_02920 | 626874 | 627221 | - | 348 |  | hypothetical protein |
| G3577_02925 | 627407 | 627754 | + | 348 |  | hypothetical protein |
| G3577_02930 | 627754 | 627996 | + | 243 |  | TIGR03758 family integrating conjugative element protein |
| G3577_02935 | 628032 | 628418 | + | 387 |  | TIGR03745 family integrating conjugative element membrane protein |
| G3577_02940 | 628431 | 628787 | + | 357 |  | TIGR03750 family conjugal transfer protein |
| G3577_02945 | 628784 | 629443 | + | 660 |  | TIGR03746 family integrating conjugative element protein |
| G3577_02950 | 629443 | 630393 | + | 951 |  | TIGR03749 family integrating conjugative element protein |
| G3577_02955 | 630383 | 631882 | + | 1500 |  | TIGR03752 family integrating conjugative element protein |
| G3577_02960 | 632258 | 632668 | + | 411 |  | TIGR03751 family conjugal transfer lipoprotein |
| G3577_02965 | 632668 | 635526 | + | 2859 |  | conjugative transfer ATPase |
| G3577_02970 | 635523 | 635900 | + | 378 |  | acetyltransferase |
| G3577_02975 | 636058 | 636726 | + | 669 |  | HNH endonuclease |
| G3577_02980 | 636719 | 637126 | + | 408 |  | hypothetical protein |
| G3577_02985 | 637131 | 637376 | + | 246 |  | hypothetical protein |
| G3577_02990 | 637570 | 637968 | + | 399 |  | TIGR03757 family integrating conjugative element protein |
| G3577_02995 | 637965 | 638957 | + | 993 |  | TIGR03756 family integrating conjugative element protein |
| G3577_03000 | 638957 | 640432 | + | 1476 | *IS5* | integrating conjugative element protein |
| G3577_03005 | 640443 | 640781 | + | 339 |  | hypothetical protein |
| G3577_03010 | 640784 | 642304 | + | 1521 | *traG* | conjugal transfer protein TraG |
| G3577_03015 | 643260 | 644273 | + | 1014 |  | helix-turn-helix domain-containing protein |
| **G3577_03020** | **644304** | **645689** | **-** | **1386** |  | **MFS** **transporter** |
| G3577_03025 | 645934 | 646851 | - | 918 | *yddG* | aromatic amino acid DMT transporter YddG |
| G3577_03030 | 647248 | 648147 | + | 900 |  | dihydrodipicolinate synthase family protein |
| G3577_03035 | 648157 | 649659 | + | 1503 |  | aldehyde dehydrogenase |
| G3577_03040 | 650039 | 650728 | + | 690 | *gntR* | GntR family transcriptional regulator |
| G3577_03045 | 650978 | 652297 | + | 1320 |  | FAD-binding oxidoreductase |
| G3577_03050 | 652579 | 653440 | + | 862 | *ISNCY* | ISNCY family transposase |
| G3577_03055 | 653443 | 653675 | + | 233 |  | hypothetical protein |
| G3577_03060 | 654122 | 654733 | + | 612 |  | hypothetical protein |
| G3577_03065 | 655779 | 656678 | + | 900 |  | DNA-binding protein |
| G3577_03070 | 656766 | 657815 | - | 1050 |  | Abi family protein |
| G3577_03075 | 658081 | 658953 | - | 873 |  | DUF3644 domain-containing protein |
| G3577_03080 | 658950 | 659600 | - | 651 |  | recombinase family protein |
| G3577_03085 | 660064 | 660432 | + | 369 |  | hypothetical protein |
| G3577_03090 | 660425 | 660799 | + | 375 |  | hypothetical protein |
| G3577_03095 | 660898 | 661263 | + | 366 |  | hypothetical protein |
| G3577_03100 | 661348 | 661995 | + | 648 |  | hypothetical protein |
| G3577_03105 | 662071 | 662673 | + | 603 |  | DUF3085 domain-containing protein |
| G3577_03110 | 662740 | 663087 | + | 348 |  | hypothetical protein |
| G3577_03115 | 663169 | 664155 | + | 987 |  | DUF1738 domain-containing protein |
| G3577_03120 | 664262 | 665188 | + | 927 |  | DUF1281 domain-containing protein |
| G3577_03125 | 665549 | 666543 | - | 995 |  | glycosyltransferase |
| G3577_03130 | 666604 | 667431 | - | 828 | *uspA* | universal stress protein |
| G3577_03135 | 667449 | 668927 | - | 1479 |  | SulP family inorganic anion transporter |
| G3577_03140 | 669352 | 669975 | + | 624 |  | recombinase family protein |
| G3577_03145 | 670030 | 673014 | + | 2985 | *tn3* | Tn3 family transposase |
| G3577_03150 | 673861 | 674046 | + | 186 | *glgS* | glycogen synthesis protein GlgS |
| G3577_03155 | 674225 | 674479 | + | 255 |  | DUF2913 family protein |
| G3577_03160 | 675311 | 675733 | - | 423 |  | hypothetical protein |
| G3577_03165 | 675987 | 676754 | - | 768 |  | glucose 1-dehydrogenase |
| G3577_03170 | 676985 | 677629 | - | 645 |  | NAD(P)H:quinone oxidoreductase |
| G3577_03175 | 677696 | 679501 | - | 1806 |  | glycoside hydrolase family 15 protein |
| G3577_03180 | 679624 | 680280 | - | 657 |  | TetR family transcriptional regulator |
| G3577_03185 | 680640 | 681320 | - | 681 |  | TetR family transcriptional regulator |
| G3577_03190 | 681510 | 683009 | + | 1500 |  | Outer membrane factor (OMF) lipoprotein |
| G3577_03195 | 683009 | 684208 | + | 1200 |  | membrane fusion component EmrA |
| G3577_03200 | 684212 | 685744 | + | 1533 |  | inner-membrane proton/drug antiporter EmrB |
| G3577_03205 | 685850 | 686791 | + | 942 | *cyoA* | cytochrome o ubiquinol oxidase subunit II |
| G3577_03210 | 686782 | 688779 | + | 1998 | *cyoB* | cytochrome o ubiquinol oxidase subunit I |
| G3577_03215 | 688769 | 689386 | + | 618 | *cyoC* | cytochrome o ubiquinol oxidase subunit III |
| G3577_03220 | 689383 | 689706 | + | 324 | *cyoD* | cytochrome o ubiquinol oxidase subunit IV |
| G3577_03225 | 689761 | 690147 | + | 387 |  | helix-turn-helix domain-containing protein |
| G3577_03230 | 690476 | 692131 | + | 1656 |  | Relaxase |
| G3577_03235 | 692188 | 693165 | + | 978 |  | site-specific integrase |
| G3577_03240 | 693376 | 693451 | - | 76 |  | tRNA-Phe |

**
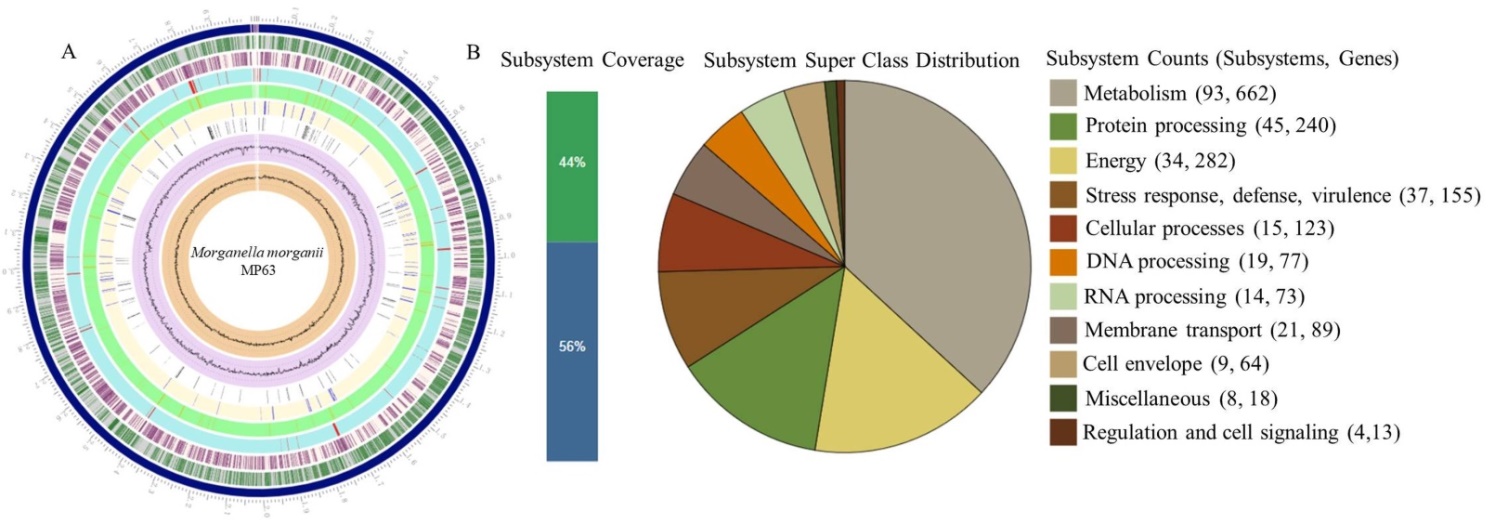
**

**Fig. S1.** Comprehensive genomic analysis of *Morganella morganii* MP63. **(A)** Circular graphical display of genomic features. From outer to inner rings: the position label (Mbp); contigs/chromosome; coding sequences (CDS) on the forward strand; CDS on the reverse strand; CDS with homology to known antimicrobial resistance (AMR) genes; CDS with homology to known virulence factor (VF) genes; CDS with homology to known transporter genes; CDS with homology to known drug targets; GC content; and GC skew. (B) An overview of the subsystems/genes found in the genome of *Morganella morganii* MP63.

**
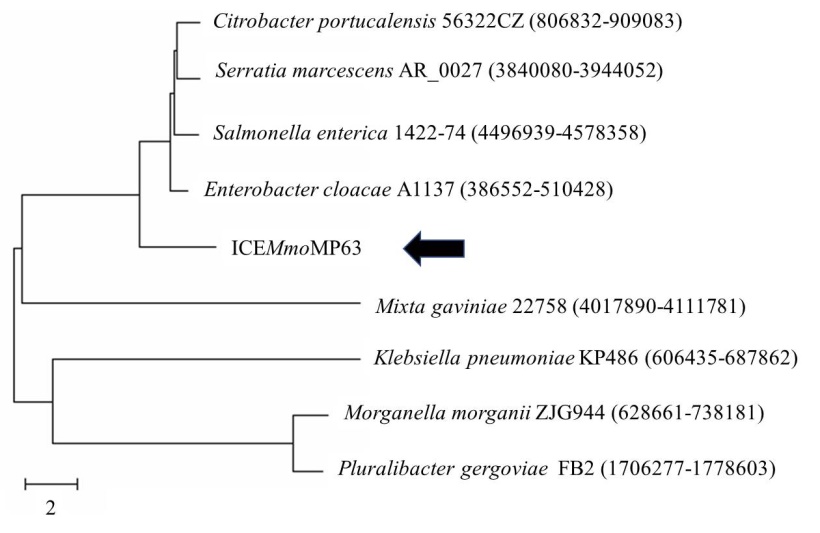
**

**Fig. S2. Phylogenetic relationships of ICE*Mmo*MP63 with mobile elements from Enterobacteriaceae bacteria.** Based on the ICE*Mmo*MP63 whole nucleotide sequence alignment results, eight mobile element sequences from different Enterobacteriaceae bacteria strains were selected, and the phylogenetic tree was constructed using MEGA7 software. The position of ICE*Mmo*MP63 in the phylogenetic tree is indicated by the black arrow.


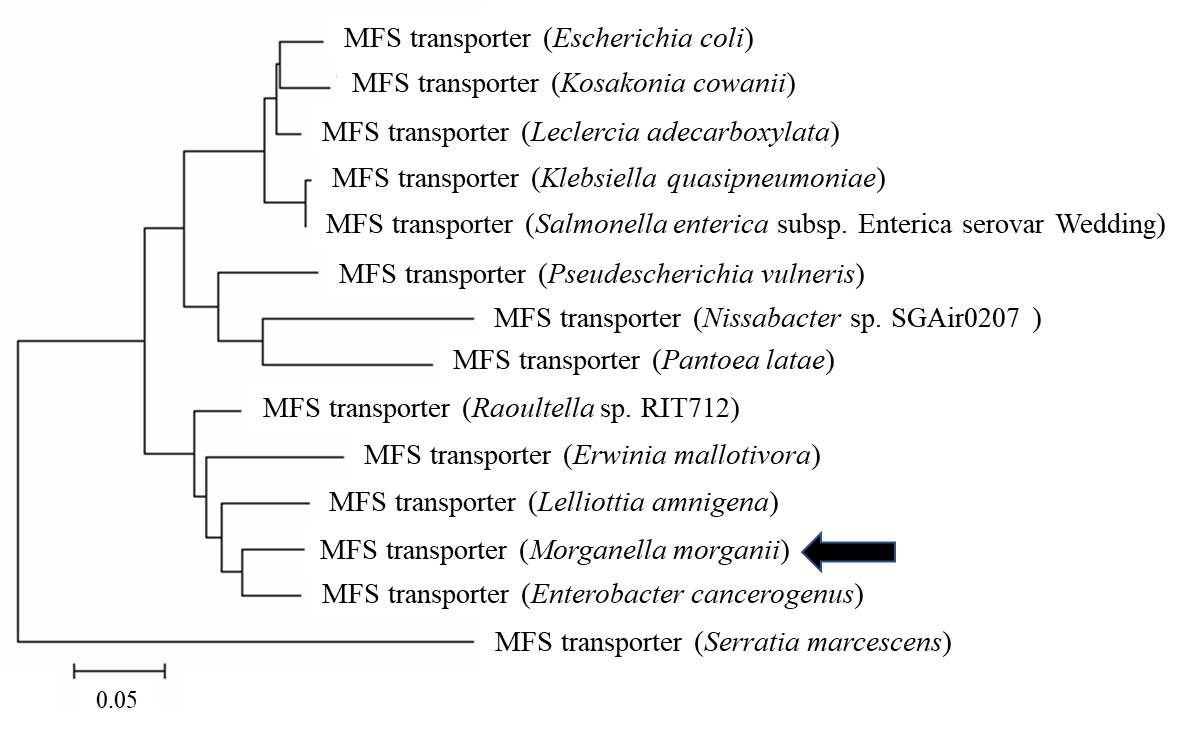


**Fig. S3.** Phylogenetic relationships of MFS transporter G3577_03020. Thirteen MFS transporter amino acids sequences from different strains were selected, and the phylogenetic tree was constructed using MEGA7 software. The position of G3577_03020 in the phylogenetic tree is indicated by the black arrow.


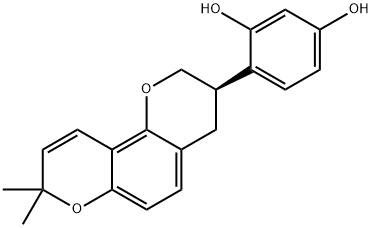


**Fig. S4.** The structure of Glabridin


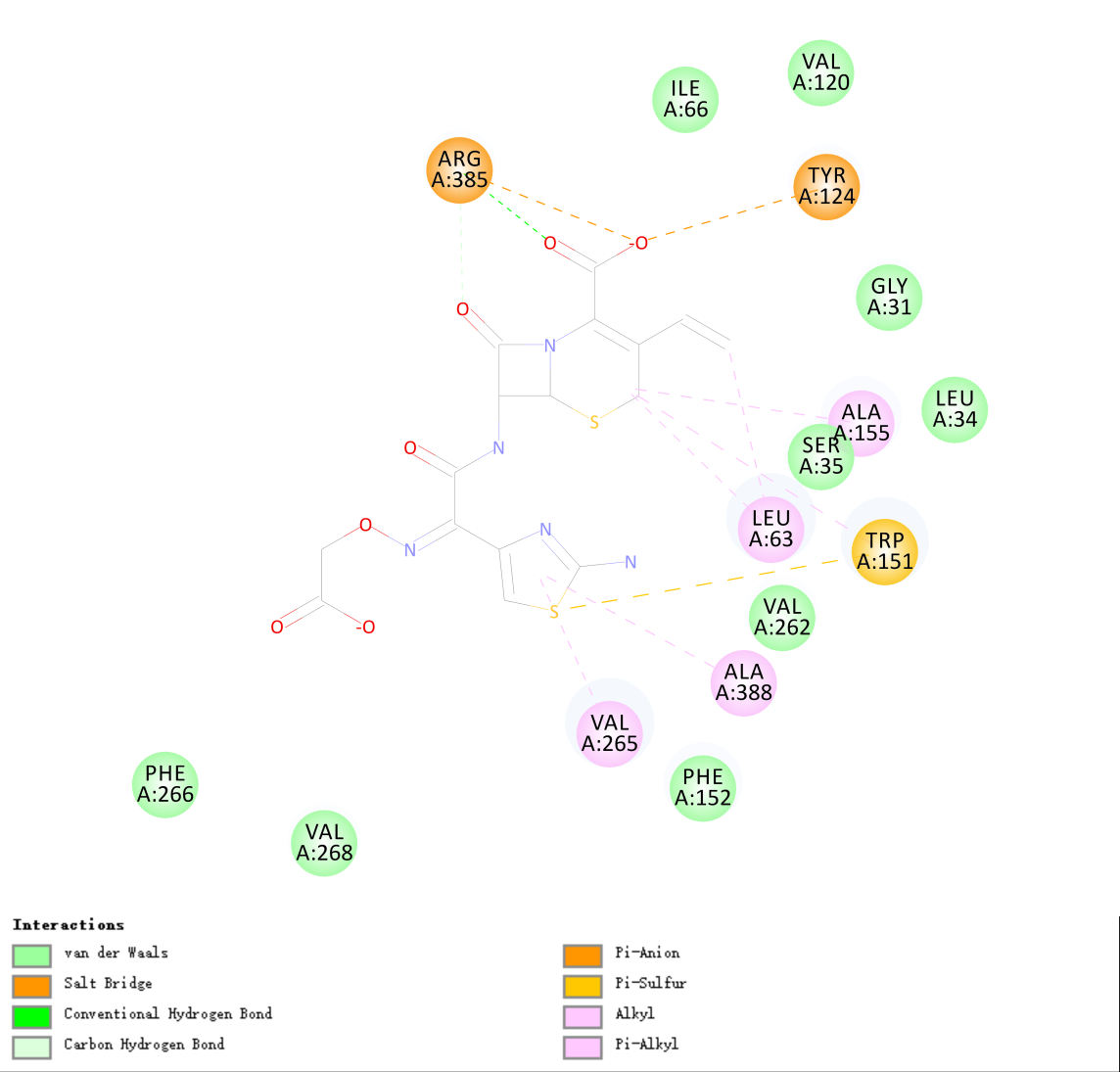


**Fig. S5. MFS transporter G3577_03020 binds with cefixime**


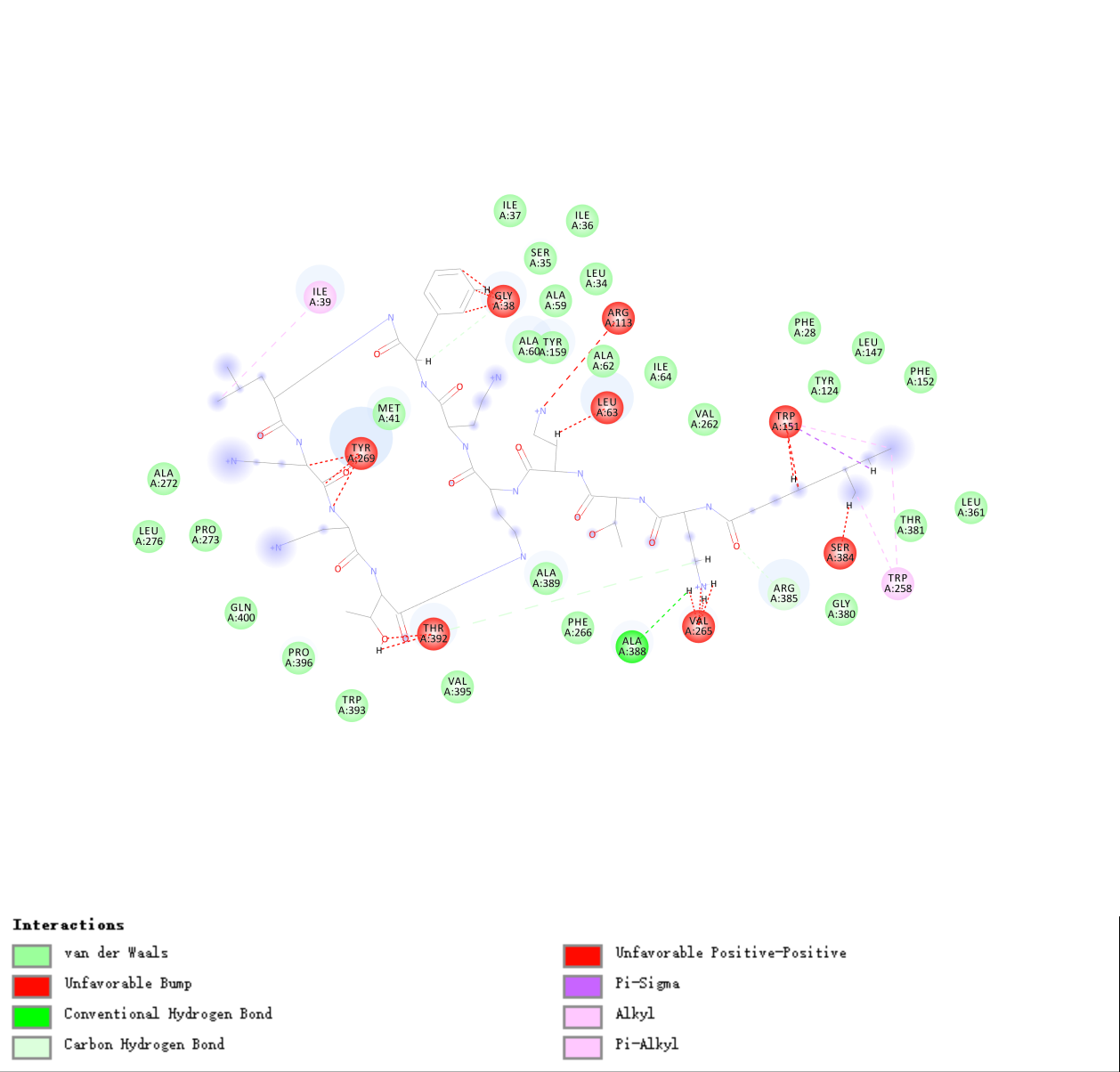


**Fig. S6. MFS transporter G3577_03020 binds with polymyxin E**


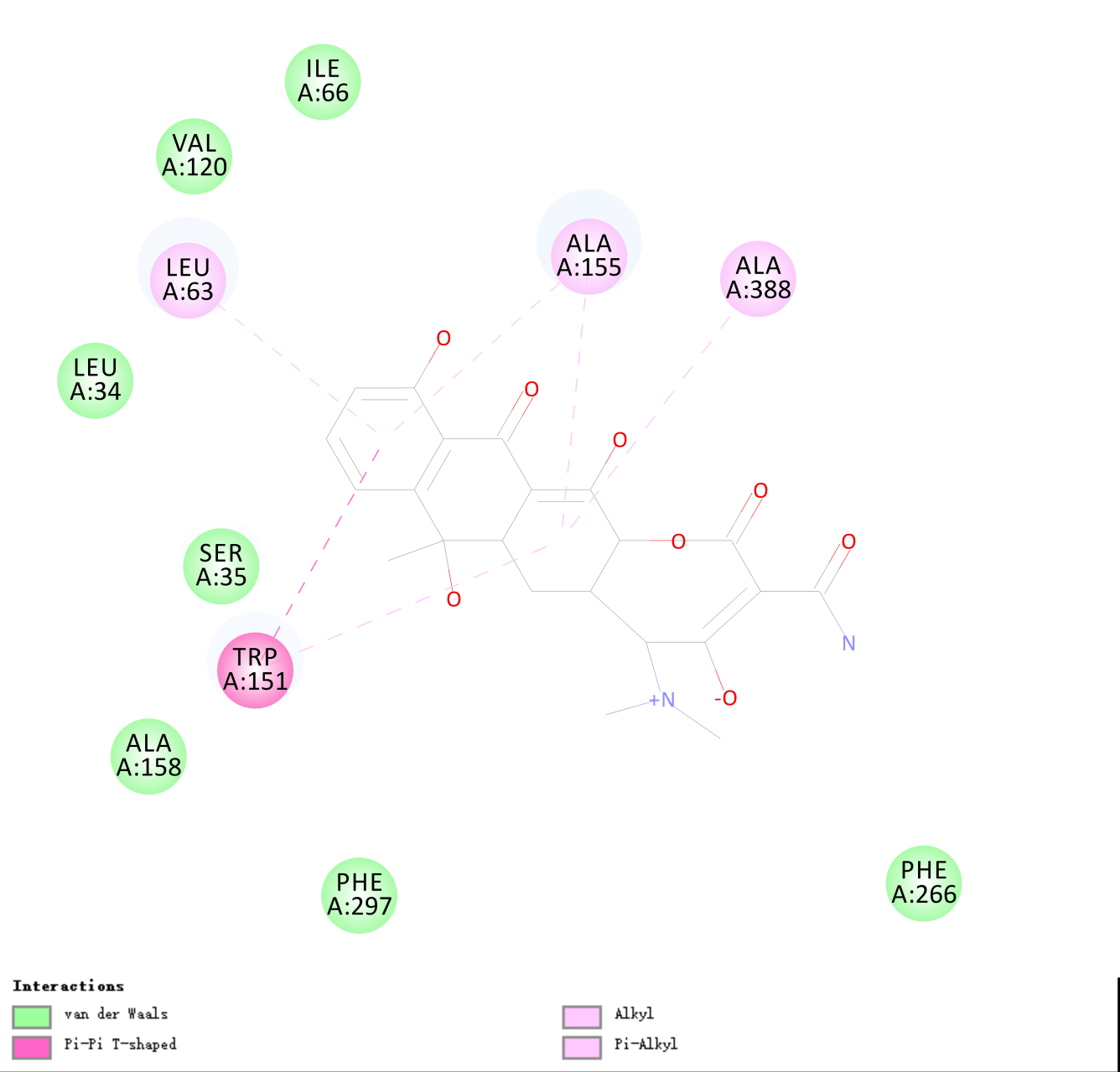


**Fig. S7. MFS transporter G3577_03020 binds with tetracycline**


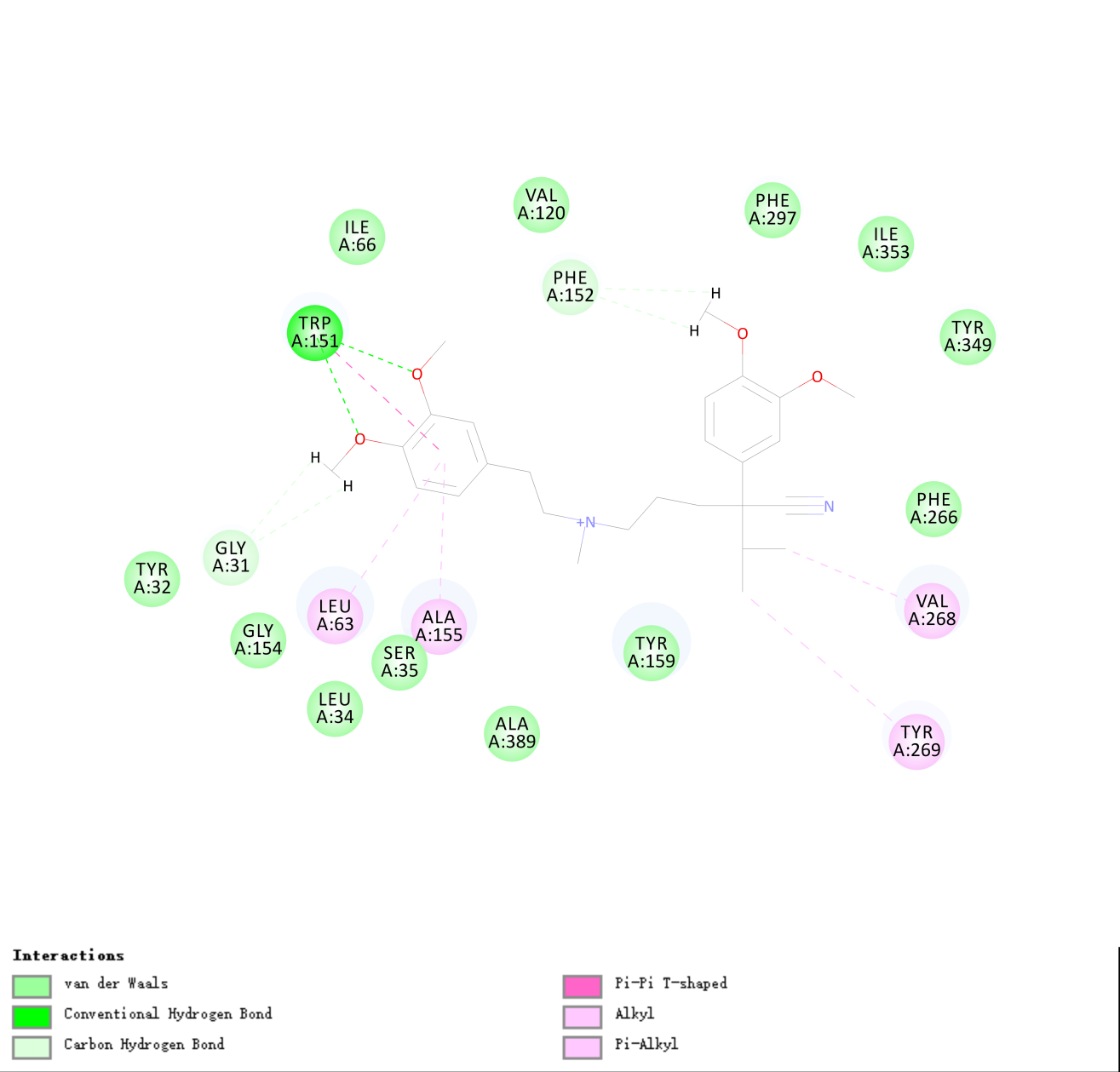


**Fig. S8. MFS transporter G3577_03020 binds with verapamil**


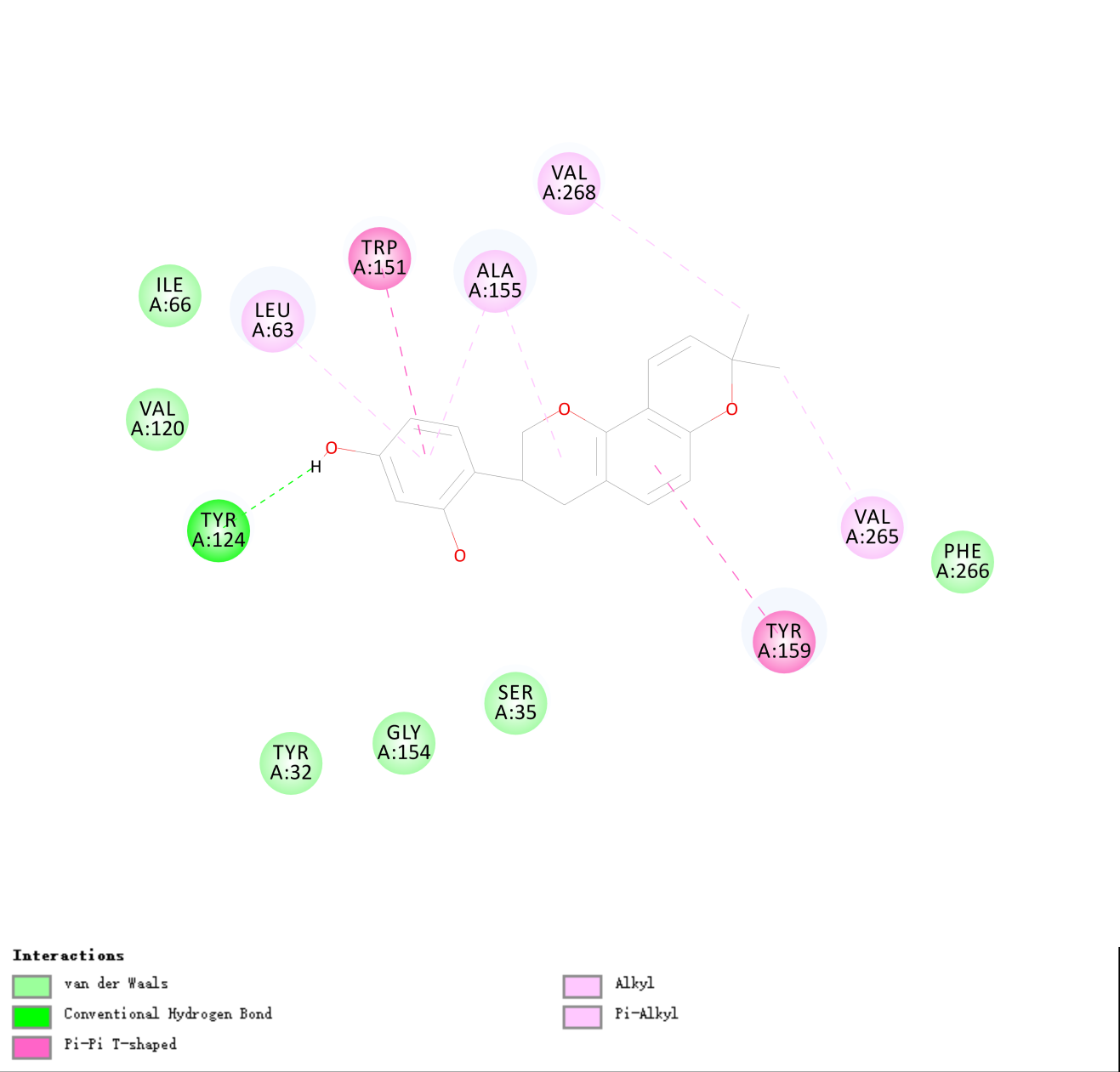


**Fig. S9. MFS transporter G3577_03020 binds with glabridin**
